# Wakefulness regulation of memory encoding and retrieval: structure and activity

**DOI:** 10.1093/nsr/nwaf520

**Published:** 2025-11-19

**Authors:** Yan-Jia Luo, Wei-Kun Su, Wei Yao, Hong Jiang, Thomas J McHugh, Ya-Dong Li

**Affiliations:** Department of Anesthesiology, Shanghai Ninth People’s Hospital, Shanghai Jiao Tong University School of Medicine, Shanghai 200011, China; School of Nursing, Shanghai Jiao Tong University, Shanghai 200025, China; Department of Gastroenterology, Songjiang Hospital and Songjiang Research Institute, Shanghai Key Laboratory of Emotions and Affective Disorders, Shanghai Jiao Tong University School of Medicine, Shanghai 201600, China; Department of Gastroenterology, Songjiang Hospital and Songjiang Research Institute, Shanghai Key Laboratory of Emotions and Affective Disorders, Shanghai Jiao Tong University School of Medicine, Shanghai 201600, China; Department of Anesthesiology, Shanghai Ninth People’s Hospital, Shanghai Jiao Tong University School of Medicine, Shanghai 200011, China; Laboratory for Circuit and Behavioral Physiology, RIKEN Center for Brain Science, Wakoshi 351-0198, Japan; School of Nursing, Shanghai Jiao Tong University, Shanghai 200025, China; Department of Gastroenterology, Songjiang Hospital and Songjiang Research Institute, Shanghai Key Laboratory of Emotions and Affective Disorders, Shanghai Jiao Tong University School of Medicine, Shanghai 201600, China

**Keywords:** wakefulness, sleep, engrams, circuits, memory, Alzheimer’s disease, Parkinson’s disease, general anesthesia

## Abstract

Sleep–wake states are fundamental regulators of memory processing. While memory consolidation relies on sleep, memory encoding and retrieval depend primarily on wakefulness. Although the role of sleep in memory consolidation has been extensively characterized, the contribution of wakefulness to memory encoding and retrieval remains less systematically summarized. In this review, we synthesize current evidence on how wakefulness regulates memory through two key dimensions: (i) structural organization, defined by the anatomical innervation of memory-related brain regions by the wakefulness system; and (ii) activity-dependent regulation, in which arousal states modulate the efficiency of memory encoding and retrieval. We highlight three major mechanisms—memory engrams, synaptic plasticity and neural oscillations—and propose adult hippocampal neurogenesis (AHN) as an additional timescale-specific mechanism linking wakefulness to memory. Finally, we discuss how wakefulness abnormalities disrupt memory encoding and retrieval in aging, Alzheimer’s disease and post-general anesthesia, and suggest that moderate enhancement of arousal level provides a novel strategy for improving memory function.

## INTRODUCTION

Mnemonic processing encompasses three steps: memory encoding, consolidation and retrieval. Sensory information processed during wakefulness allows us to perceive external stimuli and create memories, forming the foundation of memory encoding. Subsequently, these new unstable memories are thought to be strengthened off-line during sleep via processes that are key to memory consolidation. During subsequent periods of wakefulness, accessing this information can recall the stored memory and facilitate memory retrieval. Therefore, while sleep plays a critical role in memory consolidation, both memory encoding and retrieval take place during wakefulness, underscoring the importance of understanding how the circuits that drive and modulate the wake state regulate these memory processes.

Impairments of wakefulness are closely linked to memory deficits. For example, excessive daytime sleepiness (EDS) leads to impaired learning and memory recall capabilities, and is commonly observed in aging and Alzheimer’s disease (AD) populations [1]. Additionally, the inability to maintain wakefulness, as observed in cases of coma or narcolepsy, clearly results in a loss of memory acquisition abilities. Beyond these extreme conditions, variations in arousal levels impact memory. For instance, reduced arousal levels due to lack of motivation or sleep deprivation significantly impair the ability of memory encoding. Thus, in conditions such as aging, AD and post-general anesthesia, the noticeable decline in arousal level is closely linked to memory encoding/retrieval impairments [2]. Conversely, enriched environments and exercise-induced increases in arousal level are thought to benefit memory encoding/retrieval [3], although excessively high arousal levels can impair memory retrieval. However, in contrast to the extensive literature on the regulation of memory consolidation via sleep, relatively few reviews have examined how wakefulness—and the specific circuits that support it—contribute to memory encoding and retrieval.

Wakefulness is regulated by wake-promoting neurons, transmitters and neuronal circuits (collectively referred to as the ‘wakefulness system’) [4]. In this review, we outline the structural organization through which the wakefulness system regulates memory encoding and retrieval, emphasizing the activity-dependent modulation of these processes by arousal levels from three key perspectives: memory engram cells, synaptic plasticity and neural oscillations. Additionally, we propose that wakefulness regulates memory encoding/retrieval via adult hippocampal neurogenesis (AHN), which represents a novel mechanism to influence memory on a distinct timescale. Moreover, wakefulness abnormalities, as seen in aging, post-general anesthesia and neurodegenerative diseases such as AD and Parkinson’s disease (PD), are closely related to impairment of memory encoding/retrieval. Finally, pharmacological treatments, behavioral interventions and neuromodulation targeting the wakefulness system are emerging as new strategies for improving memory. Notably, this review primarily discusses how arousal regulates memory encoding and retrieval, rather than how sleep consolidates memory, as these topics have been covered well in other recent papers [5,6].

## WAKEFULNESS SYSTEM AND STATES OF WAKEFULNESS

**Figure 1.**
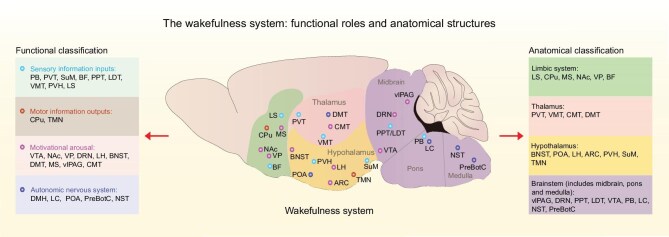
Functional and anatomical organization of wake-promoting systems. In the schematic, wake-promoting nuclei are represented by colored dots according to their primary functional and anatomical classification, as defined below. Left panel: functional classification of wake-promoting nuclei. Based on their primary roles in regulating arousal and supporting behavior, key nuclei are grouped into four interconnected functional subsystems. Sensory processing & input (blue dots): integrates and relays external stimuli to support perception and memory encoding. Includes: PB, PVT, SuM, BF, PPT, laterodorsal tegmental nucleus (LDT), ventral medial thalamus (VMT), paraventricular hypothalamus (PVH) and lateral septum (LS). Motor output & execution (orange dots): drives motor activity and exploratory behavior. Includes: caudate putamen (CPu) and TMN. Motivation & emotion (pink dots): regulates reward, salience and emotional arousal to direct goal-oriented behaviors. Includes: VTA, NAc, VP, DRN, LH, bed nucleus of the stria terminalis (BNST), dorsomedial thalamus (DMT), MS, ventrolateral periaqueductal gray (vlPAG) and central medial thalamus (CMT). Autonomic & homeostatic control (purple dots): maintains physiological stability by regulating cardiorespiratory and metabolic functions. Includes: DMH, LC, preoptic area (POA), pre-Bötzinger complex (PreBotC) and nucleus of the solitary tract (NST). Right panel: anatomical classification of wake-promoting nuclei. The same nuclei are grouped by their major brain regions for structural reference. Limbic system: LS, CPu, MS, NAc, VP, BF; thalamus: PVT, VMT, CMT, DMT; hypothalamus: BNST, POA, LH, arcuate nucleus (ARC), PVH, SuM, TMN; brainstem (midbrain, pons, medulla): vlPAG, DRN, PPT, LDT, VTA, PB, LC, NST, PreBotC. This dual classification highlights that nuclei within the same anatomical structure may serve distinct functional roles, and that coordinated activity across these functional subsystems is essential for adaptive wakeful behavior.

Wakefulness is the physiological state in which mammals exhibit full consciousness and autonomous behavior, critical for cognitive processes like attention, memory and decision-making. The brain maintains wakefulness primarily through the ascending arousal system [7], a brainstem-originating neural network that activates key brain regions via two functionally coordinated branches [4,8,9]. One branch targets the thalamus, with cholinergic input from the pedunculopontine (PPT) and laterodorsal tegmental nuclei (LDT) enhancing thalamocortical transmission and supporting alert, desynchronized cortical activity. The other branch bypasses the thalamus, directly activating the lateral hypothalamus (LH), basal forebrain (BF) and cerebral cortex; it relies on monoaminergic nuclei (e.g. locus coeruleus (LC), dorsal raphe (DR), tuberomammillary nucleus (TMN)), LH orexinergic neurons, and BF GABAergic/glutamatergic/cholinergic neurons. Above classifications of the wakefulness system are based on this framework, primarily grouping components by anatomical structure and neurotransmitter type. This anatomical and neurochemical map is essential, but it does not fully address how wakefulness supports specific brain functions. To bridge this gap, we, for the first time, propose a functional categorization that builds upon this anatomical framework. We categorize the wakefulness systems to four populations based on the distinct functions they regulate (Fig. 1).

### Sensory processing and signal relay ensembles

These deliver sensory inputs to the brain and process sensory information, including the parabrachial nucleus (PB) and LC in the brain stem [10,11], the paraventricular thalamus (PVT) [12], the supramammillary nucleus (SuM) in the hypothalamus [13] and others. Sensory inputs such as pain, cold and nausea activate neurons in the lateral PB [14–17], where glutamatergic neurons strongly promote wakefulness and relay sensory signals to the forebrain. LC norepinephrine (NE) neurons are crucial for the high arousal needed when responding to salient stimuli and stressors [11,18]. PVT glutamatergic neurons are strongly wake-promoting [12], regulating auditory, touch or tactile perception [19,20]. The SuM, a strong wake-promoting region in the posterior hypothalamus [13], delivers spatial and novelty information to the hippocampus [21,22]. In addition, the BF receives inputs from the thalamus and brainstem and contains wake-promoting neurons that deliver information to the cortex and can thus be categorized in this subpopulation [23].

### Motor output and behavioral activation ensembles

These regulate motor and behavioral actions, and include the basal ganglia, including the striatum, globus pallidus, substantia nigra (SN) and LH. Substantia nigra pars compacta (SNc) dopaminergic neurons release dopamine in the dorsal striatum, activating dopamine D1 receptor (D1R)-positive neurons to promote wakefulness [24]. Histamine promotes generalized wakefulness, as histamine levels are consistently high during wakefulness, and chemogenetic stimulation of the TMN histaminergic neurons increases locomotion [25].

### Cognitive and motivational control ensembles

These regulate higher-order brain functions, such as cognition, emotion and motivation, which rely on heightened arousal. The ventral tegmental area (VTA) dopaminergic neurons regulate motivation and reward via the ventral basal ganglia, including the nucleus accumbens (NAc) and ventral pallidum (VP) [26–28], where wake-promoting neurons, such as NAc D1R-positive neurons and VP GABAergic neurons, promote wakefulness and increase motivation [28,29]. The LH, which contains wake-promoting neurons, such as orexinergic [30], GABAergic [31], and glutamatergic neurons [32] also regulates motivation. DR 5-hydroxytrytamine (5-HT) neurons promote wakefulness related to mood [33], reward [34], patience [35] and response to salient cues [36].

### Autonomic and homeostatic regulation ensemble

This ensures that internal physiological states (e.g. cardiorespiratory function) are aligned with the metabolic demands of wakefulness and adaptive behavior. The hypothalamic paraventricular nucleus (PVN) is wake-promoting and regulates breathing [37], blood pressure [38] and heart rate [39] via the hypothalamic–pituitary–adrenal (HPA) axis. Dorsomedial hypothalamus (DMH) glutamatergic neurons regulate wakefulness [40,41], associated with breathing [42,43], blood pressure and heart rate [44]. Brainstem medullary reticular neurons and LC NE neurons also regulate wakefulness related to breathing and heart rate [45–47]. Here, we emphasize that the functional diversity of wakefulness systems is rooted in their unique anatomical projections. However, it is important to note that the categorization of the wake-promoting system based on function does not imply that they work independently. On the contrary, they are closely interconnected, ensuring functional coordination. Additionally, different functions can be integrated in one brain region, thus a single wake-promoting region may belong to multiple functional populations. Together these interactions between these different wakefulness systems form the basis for the neural network’s regulation of multiple behaviors. Their connection with memory-related brain regions and roles in memory regulation will be further elaborated in the following section.

Sleep is not a singular state; it encompasses multiple stages, including non-rapid eye movement (REM) (NREM) sleep (N1, N2 and N3) and REM sleep. Likewise, wakefulness is not uniform, as arousal levels are fluid. Based on brain sensitivity to external stimuli and cognitive processing, wakefulness can be categorized into three distinct states: drowsiness, natural wakefulness and heightened arousal, corresponding to low, medium and high arousal level, respectively [48,49]. These states are generally associated with measurable changes in brain activity, such as muscle tone, respiration, heart rate and behaviors [4,9,50]. Electroencephalograms (EEGs), which directly reflect brain activity, serve as fundamental electrophysiological indicators and the primary standard for determining arousal levels. EEG frequencies and amplitudes are classified into five bands: delta (δ, 0.5–4 Hz), theta (θ, 4–9 Hz), alpha (α, 9–14 Hz), beta (β, 14–30 Hz) and gamma (γ, 30–150 Hz) [51]. The characteristic EEG patterns during wakefulness are low-amplitude fast waves, with changes in the high-frequency α, β and γ bands closely associated with different arousal levels [52]. When transitioning from REM sleep to natural wakefulness, δ oscillations in the frontal cortex are suppressed, while α oscillations dominate in the occipital region [53,54]. As internal and external stimuli intensify, arousal level increases, marked by a decline in α oscillations and a rise in β and γ oscillations [51]. Prolonged wakefulness leads to increased slow-wave oscillation power and a gradual reduction in arousal level [55]. In addition to EEGs, observable external indicators such as pupil diameter, heart rate and behavior also reflect changes in brain states and serve as supplementary measures of arousal level. For example, pupil diameter is often used as an independent measure of arousal level [56]. Increasing arousal level by blowing air on the back of a quietly awake mouse inhibits cortical δ rhythms, enhances γ rhythms, and significantly increases pupil diameter and heart rate [57,58]. Conversely, during the transition from high to low arousal level, pupil diameter decreases [59]. A combination of these multiple indicators can comprehensively reflect differences in arousal levels.

Changes of arousal level affect cognitive performance following a classic inverted U-shaped curve [60], where both low and excessively high arousal levels are detrimental to memory. Low arousal level (e.g. drowsiness) degrades cognitive processes, including decision-making perception, conflict-handling, attentional performance and working memory [60]. However, the relationship at high arousal level is more complex. The state of ‘extreme alertness’ involves high arousal and its emotional valence (e.g. stress vs. excitement [61]), which results in memory encoding/retrieval deficits. Even in the absence of stress or emotion, sustained high arousal itself can be also harmful to memory encoding/retrieval by narrowing attentional focus, consistent with the decline phase of the inverted U-shaped model [62]. However, this effect is not absolute. Knight and Mather provided a key resolution to contradictory findings, demonstrating that the same high arousal level can enhance memory for preceding items, but only under specific conditions: when the items have high attentional weight at encoding and memory is tested after a consolidation delay [63]. Thus, hyper-arousal, regardless of valence, often pushes the system beyond its optimal point, typically impairing memory. The ensuing effect—impairment or enhancement—is determined by the complex interplay between arousal levels and time. Future research should therefore investigate the neural mechanisms through which arousal interacts with these factors to regulate cognition, moving beyond simple models to provide more nuanced insights.

## WAKEFULNESS REGULATION OF MEMORY ENCODING/RETRIEVAL: STRUCTURE AND ACTIVITY

The regulation of memory encoding and retrieval by wakefulness is primarily rooted in structural organization. All memory brain regions are innervated by the wakefulness system. For instance, the hippocampus dentate gyrus (DG) and CA1, regulating contextual and spatial memory, is influenced by wakefulness systems such as the SuM [21], BF [64], medial septum (MS) [65], LH [66], DR [67] and LC [68]. The basal ganglia form the structural basis for motor procedural memory and are regulated by the dopaminergic system from the SNc [69]. Projections from the entorhinal cortex (EC) to the hippocampus play an essential role in the regulation of episodic memory and this pathway can be modulated by the orexinergic neurons in the LH and glutamatergic neurons in the SuM [21,66]. Structural connectivity provides the foundation for wakefulness ‘being able to’ influence memory, but what determines ‘how’ wakefulness regulates memory? Arousal levels determine the efficiency of wakefulness in modulating memory processing. When the brain encounters relevant external stimuli, heightened excitability in wake-promoting regions increases arousal level. This increased excitability, together with increased arousal level, allows these wake-promoting regions to transmit stronger or selective external signals to memory-related areas, thereby facilitating memory encoding. Similarly, an elevated arousal level enhances memory retrieval by strengthening the interaction between wake-promoting regions and the specific memory circuits involved in retrieval, particularly those related to previously stored memories. Here we discuss how the arousal level regulates memory encoding/retrieval from three classic memory theories: memory engram cells, synaptic dynamics and neuronal oscillation.

### Wakefulness regulates memory through memory engrams

Memory engrams, the subset of neurons activated during memory encoding and memory recall, are often regarded as the physical representation of a memory trace. These neurons exhibit distinct characteristics: persistent changes in synaptic strength, heightened excitability upon reactivation, and a unique resting state when not actively engaged in memory processing [70–72]. Engram cells are not merely a static population, but rather dynamic entities whose activation patterns evolve over time and across multiple levels of neural organization. Wakefulness regulates these processes not as a monolithic state, but through distinct mechanisms at each level.

At the cellular level, the recruitment of neurons into engrams is primed by their intrinsic excitability—their propensity to fire action potentials [73,74]. Wakefulness promotes this by providing a neurochemical milieu (e.g. orexin, histamine) that directly biases neuronal selection. For instance, histamine, via H1 receptors in the medial entorhinal cortex (MEC), modulates neuronal excitability, and the inhibition of superficial layers of the MEC suppresses the activity of engram cells involved in memory encoding, resulting in impaired spatial learning in the Morris water maze [75]. Critically, the link between cellular excitability and ensemble fate has been directly demonstrated: a persistent increase in intrinsic excitability during learning is a key predictor determining which neurons are selectively stabilized into a long-term memory engram [76]. This maturation process is critical for understanding how wakefulness modulates their excitability and activity, especially in relation to memory stability and retrieval.

At the network level, wakefulness tunes the size and specificity of engram ensembles. Increased arousal enhances overall brain activity, influencing engram formation. For example, activation of LH orexinergic neurons during spatial memory encoding enhances recruitment in the MEC, promoting spatial memory, whereas inhibition of these projections impairs spatial memory [66]. The stability of these functionally defined ensembles is another key aspect of wakefulness regulation, as they can persist as long-term network-level templates for specific memories [77]. However, excessive arousal can lead to oversized engrams, reducing memory specificity, causing overgeneralization and impairing memory recall [78,79]. This suggests that wakefulness systems must finely tune network activity to achieve an optimal balance for distinct and stable engrams.

At the system level, wakefulness governs the brain-wide coordination necessary for engram operation. Systemic arousal increases the likelihood that engram cells are recruited and reactivated across distributed brain regions. This is evidenced by studies showing that direct activation of engram cells induces memory retrieval in diverse contexts (e.g. fear memory [80], conditioned place avoidance [81] and preference [82], inhibitory avoidance [83], object location memory [83] and social preference memory [84]), while their inhibition selectively impairs retrieval without affecting new learning [85–87]. Building on this foundational principle, a pivotal study using holographic optogenetics demonstrated that precisely reactivating a learned ensemble in the visual cortex alone can trigger a perceptual memory and elicit a conditioned behavioral response, such as licking water [88]. This highlights a key mechanism by which wakefulness, through elevated arousal level, may coordinate the faithful reactivation of specific engram ensembles to ensure efficient and accurate memory retrieval at a systems level. Accordingly, increased arousal level induced by novel environments or exercise enhances this reactivation, promoting efficient retrieval [71,89], whereas monotonous environments reduce arousal, dampen engram reactivation and impair recall.

Interestingly, different wake-promoting brain regions can similarly elevate arousal levels yet differentially influence memory encoding and retrieval. For example, LH orexinergic neurons primarily regulate memory encoding, whereas SuM neurons preferentially facilitate memory retrieval. Whether and how these regions exert distinct effects on encoding versus retrieval, and whether memory engrams are differentially engaged, remain open questions. As the concept of memory engram cells continues to advance, the next question is how to optimally enhance arousal during encoding or retrieval to either promote the formation of engram cells or selectively boost their activity during recall, thereby improving the efficiency of memory processing. Addressing this issue will be essential for deepening our understanding and for developing strategies to enhance memory function.

### Wakefulness regulates memory through synaptic plasticity

Synapses serve as the fundamental structural and functional units mediating neuronal connectivity in the brain, and activity-dependent modulation of synaptic strength (i.e. synaptic plasticity)—including long-term potentiation (LTP) and long-term depression (LTD)—is widely recognized as the cellular basis for memory formation and storage [90–94]. During wakefulness, sustained brain excitability and environmental sensory input drive the strengthening of task-relevant synaptic connections—a process essential for initial memory encoding. This wakefulness-associated synaptic potentiation is supported by studies across species—from fruit flies [95], nematodes [96] and zebrafish [97] to mice [98], with the evidence consistently showing an increase in synapse number (e.g. dendritic spine density) during prolonged wakefulness, which is essential for memory encoding. Notably, this wake-induced synaptic enhancement is not uniform but occurs preferentially in brain regions critical for memory, such as the hippocampus and prefrontal cortex, where it primes neural circuits for the acquisition of new information. In contrast, sleep plays a complementary role in refining synaptic connectivity—a process aligned with the synaptic homeostasis hypothesis (SHY) [90–99]. The SHY posits that the net increase in synaptic strength during wakefulness leads to ‘synaptic saturation,’ which could introduce background noise and interfere with subsequent memory encoding. Sleep resolves this by downscaling or pruning redundant, weak or non-task-relevant synapses (rather than reducing synapses globally), thereby restoring synaptic plasticity and ‘freeing up’ neural resources for new learning upon subsequent wakefulness. Importantly, this synaptic downregulation occurring during sleep decreases synapse density in the hippocampal CA1 region [99] and selective elimination of cortical synapses [100] across sleep episodes. Sleep also exerts region-specific effects on synaptic function: while some brain regions exhibit reduced synaptic strength post-sleep, others show stabilization of newly formed, learning-relevant synapses—for example, in the motor cortex, sleep selectively preserves dendritic spines induced by motor learning, thereby consolidating motor memories [101,102].

Several wake-promoting circuits contribute to elevated arousal states—such as those induced by environmental enrichment or exercise—and regulate memory encoding and retrieval by modulating synaptic plasticity within memory-related brain regions. LH orexinergic neurons directly modulate LTP in the MEC–hippocampal pathway, a circuit critical for spatial memory, thereby promoting the encoding of spatial information [66]. Optogenetic activation of LC–NE projections induces LTP in memory-relevant brain regions, leading to improved spatial learning and memory [103]. As a major wake-promoting nucleus with extensive hippocampal projections, activation of SuM–DG pathways enhances synaptic plasticity in the prefrontal cortex–hippocampal circuit, facilitating the retrieval of spatial memories [104]. Collectively, increased arousal level strengthens task-relevant synapses to support encoding.

### Wakefulness regulates memory through neuronal oscillation

Neural oscillations refer to the rhythmic electrical activity generated by neurons in the brain, which can be detected through local field potential (LFP) recordings or cortical EEG [105]. The wakefulness-regulating system exerts tight control over neural oscillations, and many nuclei governing wakefulness also modulate memory encoding/retrieval via oscillation-dependent mechanisms. Median raphe (MnR) serotoninergic neurons gate hippocampal theta rhythms, thereby organizing temporal coding and enabling the phase–amplitude coupling required for synaptic plasticity that supports novel-object recognition and contextual fear memory [106–109]. LC–NE projections drive sensory-evoked γ oscillations (e.g. in the hippocampus and cortex) by enhancing the excitability of pyramidal neurons; this γ enhancement directly supports fear memory encoding and retrieval via the DG [68,110]. Deletion of LH orexinergic neurons impairs the maintenance of θ and γ wave power during spontaneous wakefulness [111], while activation of LH orexinergic projections to the MEC enhances local γ power and promotes MEC–hippocampal γ coherence during spatial exploration, thereby improving spatial memory [66]. SuM glutamatergic neurons strongly modulate cortical θ and γ frequency EEG activities by regulating their spectral power, specifically increasing the spectral power of high θ(7–13 Hz) and γ oscillations when activated, and decreasing the spectral power of these oscillations when inhibited [13]. Disrupting SuM glutamate transmission to the DG abolishes hippocampal θ–γ coupling and impairs spatial memory retrieval in mice [21]. BF compromises several neuronal populations, including non-cholinergic (e.g. glutamatergic and GABAergic neurons) and cholinergic neurons. Studies have shown that their activity dynamically regulates multiple oscillation metrics; BF glutamatergic neuron activation only slightly reduces δ power and β power during slow-wave sleep (SWS), playing a weak role in cortical oscillation regulation. Activation of BF GABA neurons significantly increases high-frequency γ (60–120 Hz) power during wakefulness and reduces δ power during SWS, driving sustained wakefulness, which is critical for maintaining normal wakefulness and high-frequency oscillations. Most importantly, activation of BF cholinergic neurons suppresses low-frequency cortical oscillations (δ and θ power) during SWS and facilitates EEG desynchronization [112,113], while lesioning these neurons increases δ wave EEG activity [114], reduces wakefulness and impairs memory [115–117]. Thus, the wakefulness system biases memory by sculpting these oscillatory features—synchronization, coherence and frequency stability—within memory-critical circuits.

### Adult hippocampal neurogenesis: a novel mechanism for wakefulness regulation of memory

**Figure 2.**
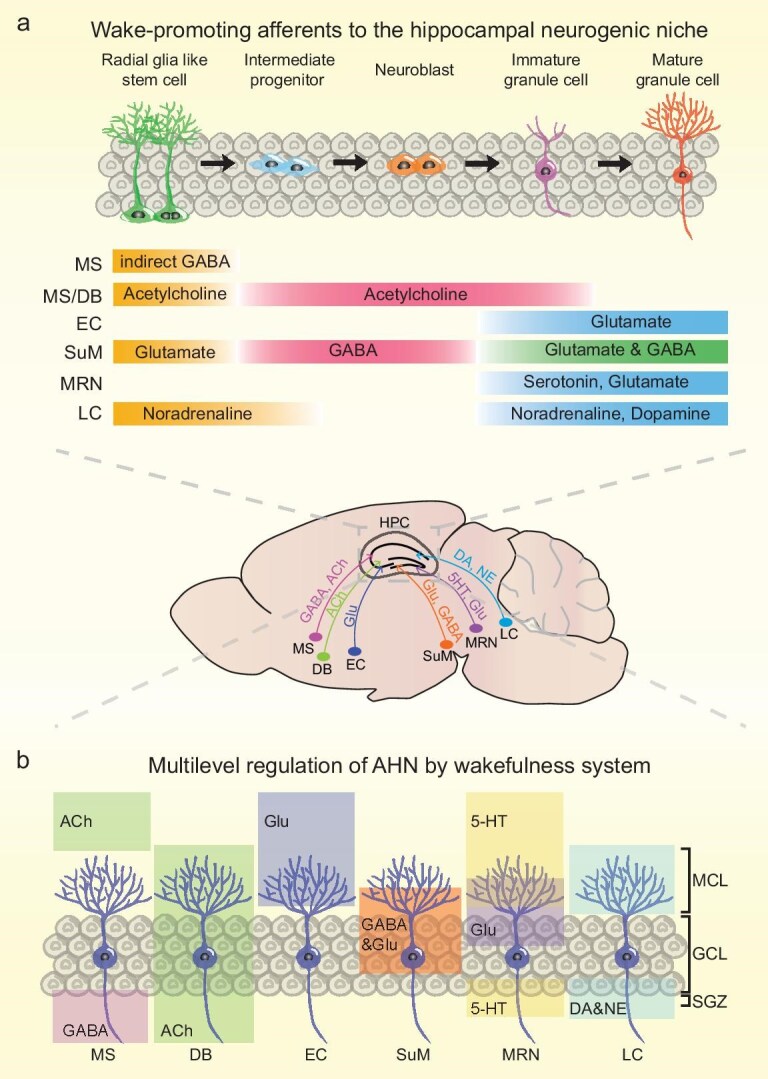
Regulation of AHN by the wakefulness system. (a) AHN is a multistage process involving radial glia‑like neural stem cells in the hippocampus, which differentiate through intermediate progenitors, neuroblasts and immature granule cells into mature granule neurons. This process is regulated by multiple arousal systems. Several wake-promoting neuronal populations project to the hippocampal neurogenic niche in the DG, including GABAergic and cholinergic neurons from the MS, cholinergic neurons from the diagonal band (DB), glutamatergic neurons from the EC, GABA/glutamate co-releasing neurons from the SuM, serotonergic and glutamatergic neurons from the median raphe (MRN), and dopaminergic and noradrenergic neurons from the LC. The various neurotransmitters released by distinct wakefulness circuitry influence different stages of AHN, including neural stem cells (NSCs), progenitors, neuroblasts and immature/mature neurons. These processes regulate NSC proliferation, differentiation, survival and maturation. Ultimately, the newborn neurons are integrated into hippocampal circuits, where they play a crucial role in regulating memory function. (b) These inputs of wakefulness system release neurotransmitters (GABA, acetylcholine, glutamate, serotonin, NE and DA) within the DG, modulating distinct stages of AHN. These neurotransmitters act on specific subcellular compartments (e.g. cell bodies, dendrites, axons) of developing ABNs and local interneurons, thereby fine-tuning their excitability, synaptic plasticity and circuit integration. This multilevel regulation of AHN by wakefulness-related circuits not only shapes local hippocampal microcircuits but also impacts brain-wide network dynamics, ultimately influencing memory precision, contextual discrimination and emotional behavior.

Neurogenesis primarily occurs during embryonic, early childhood and adolescent stages, with limited occurrences in adulthood. Nonetheless, at each stage, neurogenesis closely correlates with memory. Impairment of neurogenesis in young mice can impair adulthood learning and memory abilities [118]. In the adult brain, only specific regions like the subgranular zone (SGZ) of the hippocampus continue to produce a small number of newborn neurons, which integrate into the existing hippocampal circuits to regulate memory [119] (Fig. 2a). The regulation of memory by AHN is a multidimensional process, wherein the synergistic enhancement of adult newborn neuron number [120,121], activity [122] and maturation state is crucial for memory improvement, while AHN deficiencies can lead to memory impairment [123–128].

The multilevel wakefulness system regulates AHN (Fig. 2b), which allows moderate increases in arousal level by enriched environments or exercise promoting AHN. The SuM, as a key wake-promoting region, delivers novelty information in the enriched environment to the DG and thus promotes AHN by co-releasing γ-aminobutyric acid (GABA) and glutamate, and affects different stages of AHN. Interestingly, further activity regulation of these SuM-enhanced adult-born neurons (ABNs) enhances spatial and contextual memory retrieval by increased hippocampal circuitry activity and synaptic plasticity [123,129,130]. These studies provide direct evidence that the wakefulness system regulates AHN, thereby contributing to memory retrieval and linking this process to other classical mechanisms of memory regulation. Furthermore, orexin released by LH neurons boosts AHN in the DG, promoting the differentiation of progenitor cells into neurons [131]. Additionally, other wake-promoting neurotransmitters, such as NE, dopamine and 5-HT, influence the proliferation and differentiation of neural stem cells through different pathways [132,133], thereby affecting memory. In AD mice, decreased arousal levels and impaired AHN are associated with memory deficits [134], while activation of the SuM in early AD brains can still promote AHN and improve memory retrieval [129]. Distinct from the immediate mechanisms of engrams, synaptic plasticity and neural oscillations on memory regulation, the influence of the wakefulness system on AHN operates on a much longer timescale. AHN persists for weeks in rodents, and years in humans, suggesting that the wakefulness system can exert prolonged effects (spanning months to years) on memory encoding and retrieval by modulating the generation and integration of ABNs in the hippocampus. The time scale is substantially longer than that of other mechanisms.

**Figure 3.**
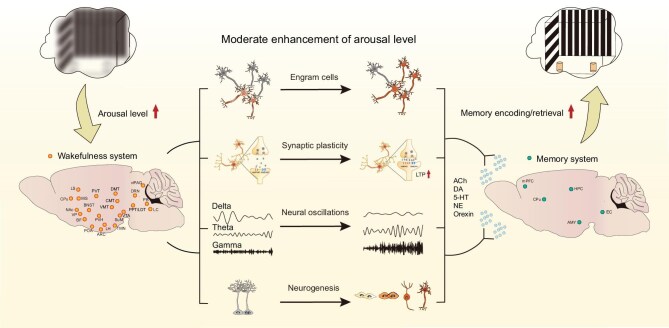
Wakefulness regulation of memory encoding and retrieval. The structural basis of the wakefulness system’s influence on memory regions, along with the activity-dependent regulation of memory by arousal levels, collectively underpins wakefulness-mediated memory regulation. Four key aspects—memory engram cells, synaptic plasticity, neuronal oscillations and AHN—are central to this process. The efficacy of these processes is non-linearly dependent on arousal level. Low arousal levels impair memory encoding and retrieval, while increasing arousal levels within an optimal level enhances them by ensuring precise neuromodulatory control.

In summary, a moderate increase of arousal level regulates memory encoding and retrieval through influencing memory engram cells, synaptic plasticity, neural oscillations and AHN (Fig. 3). These mechanisms are interrelated rather than independent. For instance, ABNs exhibit higher synaptic plasticity than mature granule cells or pyramidal neurons in the hippocampal circuits. The activity of memory engram cells and neural oscillations mutually influence each other in memory regulation. Understanding how these mechanisms integrate and synergize remains a central focus of research on wakefulness and memory regulation in the foreseeable future. Disentangling these aspects remains challenging, but several experimental approaches can help determine whether a wake-promoting system directly regulates memory. Is the activity of wake-promoting neurons that project to memory regions modulated by memory-related behaviors? Does direct manipulation of wakefulness system projections specifically within memory-related brain regions alter memory encoding or retrieval? Are there any specific physiological or pathological contexts that can alter activity of the wakefulness system simultaneously affecting memory and arousal levels?

## WAKEFULNESS AND PERIOPERATIVE NEUROCOGNITIVE DISORDERS

Perioperative neurocognitive disorders (PNDs) encompass acute and long-term cognitive impairments following anesthesia and surgery, including delirium in the immediate postoperative period, and postoperative cognitive dysfunction. The decline in memory and concentration observed in patients after surgery can persist for weeks to months [135,136]. This condition manifests as an inability to recall events that occurred from the induction of general anesthesia until several hours after awakening, as well as a long-term decline in memory. General anesthetics exert their effects on the brain by broadly inhibiting the activity of most neurons, including those involved in the wakefulness system. Consequently, these anesthetics may persistently suppress the wakefulness system, reduce arousal levels and lead to postoperative memory impairment. In this section, we discuss how general anesthetics, such as sevoflurane and propofol, inhibit the wakefulness system, resulting in memory encoding and retrieval deficits.

### Differences between recovery from general anesthesia and physiological wakefulness

**Figure 4.**
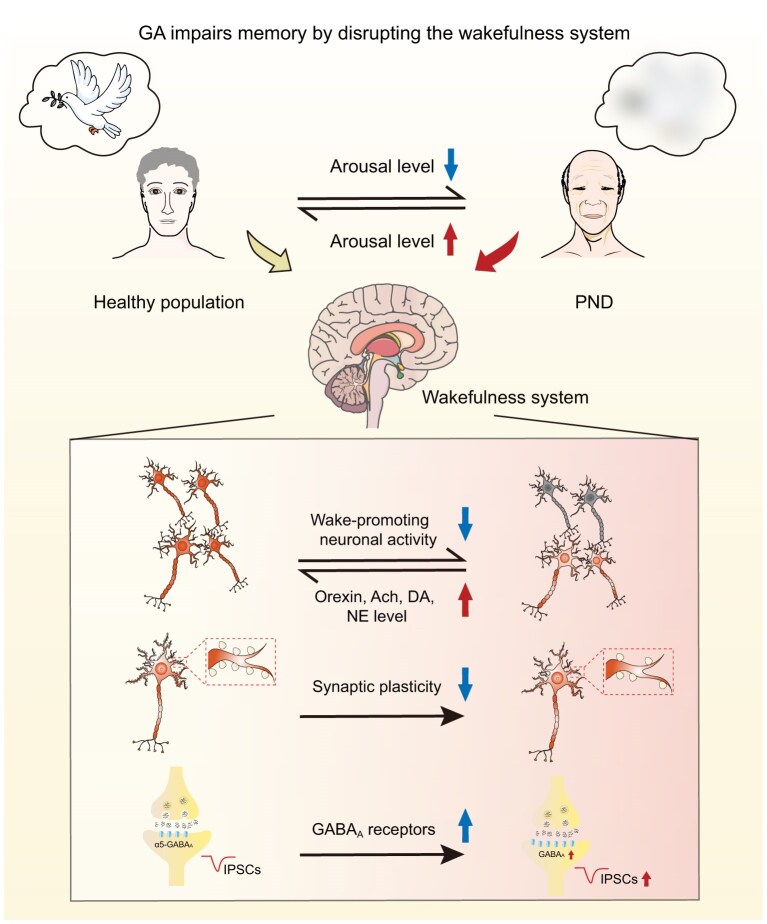
General anesthesia impairs memory by decreasing arousal levels. General anesthetics induce a sustained suppression of the wakefulness system through multiple mechanisms: inhibition of wake-promoting neurons, impaired synaptic plasticity and enhanced GABAergic inhibition. These effects synergistically exacerbate underlying vulnerabilities in patients with aging or neurodegenerative disorders, significantly increasing the risk of memory deficits. Moderate enhancement of arousal level can improve memory after general anesthesia via above pathways. GA: general anesthesia.

Recovery from general anesthesia does not equate to a return to physiological wakefulness. While both sleep and anesthesia involve a reduction in information integration and processing, there is a key difference in how the brain’s activity is altered. During sleep, cortical activity decreases uniformly, whereas anesthesia causes uneven inhibition across different brain regions. This is especially evident in the disrupted connectivity between cortical areas and subcortical regions [137], highlighting the unique impact of anesthesia on brain function. Unlike physiological arousal, during recovery from anesthesia, EEG activity in the brain returns quickly, but the recovery of cognitive function is a gradual and delayed process [138–140]. The return of consciousness and cognitive abilities following anesthesia is not entirely aligned with the metabolic clearance of anesthetic agents from the body. Increasing evidence suggests that anesthetics can continue to exert inhibitory effects on certain brain regions even after anesthetics have been metabolized and cleared. Molecularly, etomidate and isoflurane increase the expression of α5-GABA_A_ receptors, resulting in sustained inhibitory currents lasting nearly a week [139]. Propofol decreases the expression of KCC2 in the ventral posterior medial (VPM) nucleus, increasing intracellular chloride concentration and leading to GABA_A_ receptor-mediated depolarization, which promotes the loss of consciousness [141]. Dopamine reuptake in the NAc remains blocked for 4 h after propofol anesthesia, contributing to an antidepressant effect [142]. In the developing brain, early exposure to general anesthetics such as midazolam and isoflurane can lead to detectable synaptic loss within hours and, importantly, these changes in synaptic structure can persist into adulthood [143]. On the cellular level, in the hippocampal CA1 region, anesthesia induces a marked suppression of local field potentials and calcium activity that is more profound than during natural sleep and can linger for up to 6 h [144]. The prolonged suppression of receptor, synaptic and neuronal activity is a key mechanism underlying postoperative wakefulness and memory impairments (Fig. 4).

### Impact of general anesthesia on specific wakefulness system and memory

General anesthesia influences brain regions and neural pathways involved in both wakefulness and memory (Table 1), contributing to memory impairment by directly affecting neuronal activity in regions critical for memory processing, such as the amygdala, hippocampus, prefrontal cortex and EC. Despite recovery of neuronal activity after anesthesia, memory deficits often persist for extended periods [138], suggesting that prolonged suppression of wake-promoting neurons may contribute to these lasting cognitive impairments. For instance, isoflurane inhibits BF GABAergic neurons, whereas activation of these neurons promotes behavioral and cortical emergence from isoflurane anesthesia [145]. LC noradrenergic neurons project to the DG, amygdala and CA1 region, playing a role in the regulation of contextual and fear memory [68,146,147] and are inhibited specifically by isoflurane during general anesthesia [148]. VTA dopaminergic neurons, which are also inhibited by isoflurane [149], project to the hippocampus and prefrontal cortex to promote contextual learning [150,151]. Orexinergic neurons from the LH project to the hippocampus and EC to regulate spatial memory encoding [66,152,153] and show sensitivity to multiple typical anesthetics [154–156]. Furthermore, both DR nucleus (DRN) serotonergic neurons and PVT glutamatergic neurons project to the basal amygdala to regulate aversive memory encoding [157]. DRN serotonergic neurons are inhibited under isoflurane anesthesia [158], whereas PVT glutamatergic neurons are inhibited by propofol [159]. Likewise, glutamatergic neurons in the lateral PB, which project to the central amygdala to regulate fear memory [160], are inhibited under sevoflurane anesthesia [161]. Further investigation into how specific anesthetics, such as sevoflurane and propofol, differentially impair distinct wake-promoting systems will clarify the mechanisms through which general anesthesia selectively disrupts memory encoding and retrieval.

**Table 1.** Impact of general anesthesia on wakefulness system and memory.

Wakefulness system	Neuron type	Anesthetics	Projections to memory regions	Memory
BF	Cholinergic	Isoflurane [162] Propofol [162,163]	HPC Cortex AMY	Spatial memory [164] Emotional memories [165]
	GABAergic	Isoflurane [145]	Cortex	Episodic memory [166]
	Glutamatergic	Propofol [167]	AMY Cortex	N/A
MS	Cholinergic	Isoflurane [168,169] Propofol [169]	HPC ACC	Episodic memory [170]
diLS	GABAergic	Isoflurane [171]	N/A	N/A
BNST	GABAergic	Isoflurane [172]	AMY	Fear memory [173,174]
NAc	GABAergic D1R-expressing	Sevoflurane [175]	N/A	Cocaine-associated memory [176]
VP	GABAergic	Propofol [177]	NA	Fear memory [178]
PVT	Glutamatergic and calretinin-expressing	Sevoflurane [179] Propofol [159]	CeA	Fear memory [180]
CL	Glutamatergic	Isoflurane, propofol [181]	N/A	N/A
TMN	Histaminergic	Isoflurane [182]	MEC NAc	Social and object recognition memory [183]
	GABAergic	Sevoflurane [184] Propofol [184]	N/A	N/A
PVH	Glutamatergic	Isoflurane [185]	N/A	N/A
LH	Glutamatergic	Isoflurane [186]	N/A	Morphine-withdrawal memory [187]
	Orexin/hypocretin-expressing neurons	Propofol [154] Desflurane [155] Isoflurane [156]	HPC MEC	Spatial memory [66]
LC	Noradrenergic	Isoflurane [188] Propofol [189]	DG CA1 AMY	Episodic memory and fear memory [68,146,147]
DRN	Serotonergic	Isoflurane [158] Sevoflurane [190]	Basal amygdala	Aversive Memory [157]
VTA	Dopaminergic	Isoflurane [149] Sevoflurane [191] Propofol [192]	PFC HPC	Contextual learning [150,151]
vPAG	Dopaminergic	Propofol [193] Isoflurane [194]	AMY	Fear memory [195]
PPT	Glutamatergic	Sevoflurane [196]	N/A	N/A
PB	Glutamatergic	Sevoflurane [161] Isoflurane [197]	CeA	Fear memory [160]
NST	GABAergic	Propofol [198]	N/A	N/A
LHb	Glutamatergic	Isoflurane [199] Propofol [200]	HPC	Spatial memory [201]
DMH	Glutamatergic	Isoflurane [202]	AMY	Fear memory [203]
OT	Dopaminergic	Isoflurane [204]	N/A	Associative memory [205]
mPFC	Glutamatergic	Isoflurane [206]	AMY	Fear memory [206] Episodic memory [207] Cocaine-associated memories [208]
	Cholinergic	Sevoflurane [209]	N/A	Working memory [210]
	Dopaminergic	Propofol [211]	N/A	Working memory [212]

AMY, amygdala; BNST, bed nucleus of the stria terminalis; CeA, central nucleus of the amygdala; CMT, centromedian thalamic nucleus; CL, central lateral thalamus; diLS, dorsal-intermediate lateral septum; HPC, hippocampus; LHb, lateral habenula; MS, medial septum; mPFC, medial prefrontal cortex; NST, nucleus of the solitary tract; OT, olfactory tubercle; PFC, prefrontal cortex; PVH, paraventricular nucleus of the hypothalamus; PB, parabrachial nucleus; PVT, paraventricular thalamus; vPAG, ventral periaqueductal gray; vCA1, ventral hippocampal CA1.

## WAKEFULNESS AND MEMORY IMPAIRMENTS IN PATHOLOGICAL CONDITIONS

**Figure 5.**
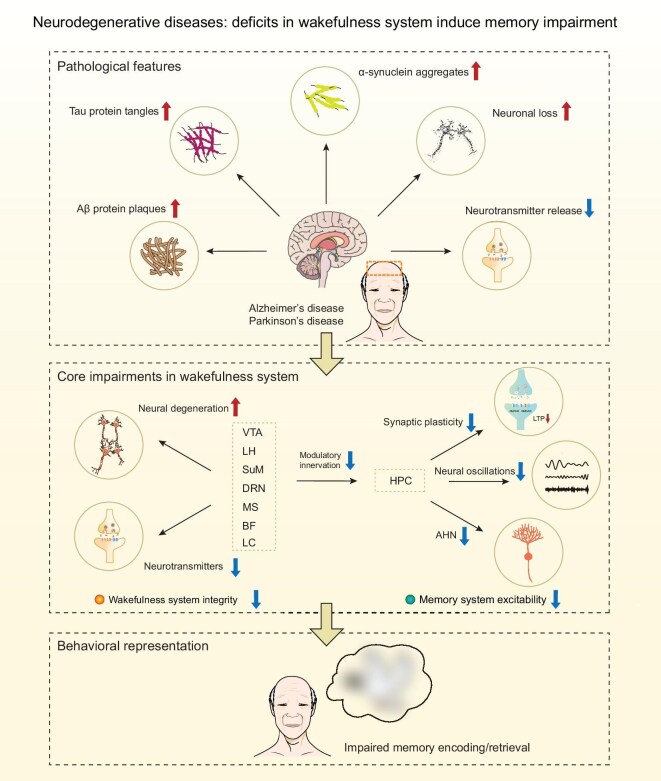
The impaired wakefulness system represents a crucial, but underrecognized, link between neurodegeneration and cognitive decline. In neurodegenerative diseases such as AD and PD, pathological hallmarks—including amyloid-β plaques, tau tangles, α-synuclein aggregates, neuronal loss and diminished neurotransmitter release—disrupt the wakefulness system. Vulnerable nuclei, such as the LC, BF, SuM, LH, VTA, DR and MS, undergo early degeneration, leading to reduced excitatory and modulatory input to memory-related regions. Mechanistically, dysfunction in these wake-promoting circuits compromises key substrates of memory encoding and retrieval. This dysfunction impairs the ability to form and reactivate memory engrams, decreases synaptic plasticity (e.g. long-term potentiation), disrupts neural oscillations and suppresses AHN. Collectively, these alterations weaken engram formation and reactivation, degrade circuit synchrony and limit structural plasticity, ultimately contributing to progressive deficits in memory encoding and recall in both AD and PD. Thus, impairment of the wakefulness system represents a crucial, but underrecognized, link between neurodegeneration and cognitive decline. Targeting the wakefulness system to increase arousal level provides promising avenues for mitigating memory dysfunction in neurodegenerative disease.

Sleep plays a crucial role in the process of memory consolidation, and the impact of sleep disorders on cognitive impairments associated with neurodegenerative diseases has been extensively studied [213–216]. Importantly, impairments in daytime wakefulness emerge even earlier than sleep disturbances in AD and are strongly associated with deficits in memory encoding and retrieval [1,217]. Despite this, the role of wakefulness abnormalities has received comparatively less attention. In this section, we review how impairments in wakefulness contribute to memory encoding and retrieval in aging and pathological conditions such as AD and PD (Fig. 5).

### Wakefulness and memory impairments in aging

The prevalence of EDS and daytime napping increases with advancing age [213,218]. EDS has been identified as a risk factor for long-term cognitive decline [217] and all-cause dementia [219,220]. The occurrence of EDS reflects dysfunction in the wakefulness system, as indicated by the decreased power and frequency of waking α oscillation with advancing age, which subsequently impairs cognitive abilities [221–223]. The integrity of the LC correlates positively with episodic memory performance in older adults [224] and aging associated with declines in LC function impair emotional memory [225]. Similarly, a gradual decrease in dopamine D2 receptors in the striatum [226] with aging is negatively correlated with declines in working memory and episodic memory [227]. The BF–cholinergic system also undergoes functional decline during aging, characterized by reduced cholinergic fiber projections to the hippocampus and cortex. This reduction leads to decreased amplitude and frequency of hippocampal θ oscillations, as well as reduced temporal precision of hippocampal γ oscillations coupled with specific θ phases, thereby affecting associative memory [228]. Importantly, aging is a natural biological process, thus the impairment of the wakefulness system in aging differs from that in neurodegenerative diseases such as AD and PD. In AD and PD patients, general anesthesia suppresses the wakefulness system and exerts prolonged inhibitory effects, which could further impair arousal levels in patients, potentially leading to severe PND.

### Wakefulness and memory impairments in AD

Neurodegeneration within key memory regions such as the hippocampus is widely recognized as a primary cause of memory impairment in AD. However, dysfunction of wakefulness systems emerges earlier than hippocampal degeneration and may significantly contribute to cognitive decline. Behaviorally, memory impairment in AD patients is associated with early onset and progressive worsening of insufficient arousal as the disease advances [1,229,230]. Post-mortem studies have shown significant loss of wake-promoting neurons in the LC, LH and TMN in AD patients [2]. In particular, the volume of the LC decreases progressively with the progression of AD [231]. In AD mouse models, tau protein deposition in the LC impairs the regulatory capacity of dopaminergic and noradrenergic systems over hippocampal plasticity, leading to memory decline [232]. Loss of VTA dopaminergic neurons affects synaptic plasticity in hippocampal CA1 and dopamine release in the NAc, resulting in memory deficits in AD mouse models [233,234]. Deposition of Aβ protein in the BF disrupts signaling through nicotinic acetylcholine receptors, thereby impairing cholinergic signaling, acetylcholine synthesis, release and transport, and compromising long-term potentiation in the hippocampus, affecting memory encoding [235]. Chemogenetic activation of cholinergic projections from the MS to the hippocampus improves spatial memory in amyloid precursor protein (APP) transgenic mice [164]. Loss of DR serotonergic neurons and projections to the septum and hippocampus impairs AHN and memory [236]. The SuM is involved in perceiving and responding to novel stimuli, selectively transmitting stimulus information to the DG and hippocampal CA2, thereby participating in memory processes [237]. Our recent work has demonstrated that SuM activity declines in the early stages of AD, and that targeted stimulation of SuM–DG projections can directly rescue memory retrieval in AD mice [22].

Orexinergic neurons in the LH are crucial in regulating both memory and wakefulness, and their functional changes in AD present contradictory results. Loss of orexinergic neurons has been reported in AD patients [238]; however, orexin levels in the cerebrospinal fluid (CSF) of AD patients show an increasing trend [239]. This increase leads to nocturnal wakefulness and fragmented sleep, potentially causing EDS and subsequent cognitive decline [239,240]. Deleting the *orexin* gene in AD mouse models reduces brain Aβ deposition as sleep duration increases [241]. Current research suggests that overactivation of orexinergic neurons during sleep contributes to AD progression by reducing sleep efficiency and shortening sleep duration [238–240]. Yet, the functional changes of orexinergic neurons during the AD process and their interaction with wakefulness and memory impairment require further investigation.

### Wakefulness and memory impairments in PD

EDS and memory impairment are recognized features of PD. EDS tends to worsen as PD progresses, with a higher prevalence observed in PD patients with dementia [242,243]. Dopamine depletion in the basal ganglia and degeneration of dopaminergic neurons are typical pathological characteristics of PD. PD mouse models injected with 6-hydroxydopamine (6-OHDA) into the medial forebrain bundle show significant loss of dopamine neurons in the SNc and VTA, along with a marked reduction in dopaminergic fiber projections in the dorsal striatum and VP, resulting in deficits in active wakefulness [244,245]. It has been confirmed that striatal D1R-positive neurons are involved in the initiation and maintenance of arousal [24]. PD patients with EDS exhibit more severe presynaptic dopaminergic dysfunction in the striatum compared to those without EDS [246]. Reductions in dopamine content in the dorsal striatum and loss of dopaminergic signals can lead to decreased spatial working memory and cognitive flexibility in PD mice [247]. PD is also characterized by loss of cholinergic neurons, and cognitive decline is associated with dysfunction of the cholinergic system [248]. Accumulation of α-synuclein and atrophy of BF cholinergic neurons leads to impaired memory [249]. PD involves loss of orexinergic neurons and decreased levels of orexin in CSF [250]. Chemogenetic activation of LH orexinergic neurons in PD mouse models has been shown to improve their social memory [251].

## TREATMENT OF MEMORY IMPAIRMENTS BY TARGETING THE WAKEFULNESS SYSTEM

Currently, the treatment of memory impairment in neurodegenerative diseases lacks specific strategies. However, some pharmacological and non-pharmacological interventions targeting the wakefulness system have shown preliminary efficacy in improving memory, offering new strategies for addressing memory impairments.

### Pharmacological interventions

Modafinil is a potent wakefulness-promoting agent that affects multiple arousal systems. It not only treats narcolepsy but also improves memory function in patients with sleep deprivation [252,253]. Modafinil acts on LH orexinergic neurons and promotes spatial memory by regulating γ oscillations and glutamatergic synaptic plasticity in the MEC [66]. More importantly, modafinil has been found to improve attention and emotional blunting in AD patients. Additionally, oveporexton, another orexin receptor 2-selective agonist used to treat narcolepsy, can also increase wakefulness and potentially improve memory encoding in AD [254]. Methylphenidate hydrochloride is a central nervous system stimulant that inhibits the reuptake of NE and dopamine in presynaptic neurons, thereby increasing their concentration in the synaptic cleft, and improving cognitive function in AD patients [255]. Donepezil, a selective cholinesterase inhibitor, has become a first-line treatment for cognitive impairment in AD [256,257]. Another cholinesterase inhibitor, rivastigmine, has been shown to improve cognitive deficits in PD by enhancing cholinergic neurotransmission in the BF, thereby improving verbal and spatial memory abilities [258]. By targeting the wakefulness system, pharmacological interventions hold promise for improving memory in patients with neurodegenerative diseases.

### Behavioral interventions

Cognitive training is an intervention method that employs various cognitive tasks to enhance cognitive functions. By utilizing systematically designed tasks, cognitive training targets cognitive domains such as attention, memory and logical reasoning, providing difficulty-adaptive training based on synaptic plasticity. This approach improves cognitive functions by increasing the information input to patients [259]. Exercise can promote the release of arousal-related neurotransmitters such as NE, 5-HT and acetylcholine, as well as hippocampal brain-derived neurotrophic factor (BDNF), which supports synapse formation [260]. Studies have shown that aerobic exercise can reduce the risk of developing AD and improve cognitive functions in AD and PD patients [261,262].

### Neuromodulation

Non-invasive neuromodulation techniques have been used to improve cognition in patients with neurodegenerative diseases. Repetitive transcranial magnetic stimulation (rTMS) can increase cortical excitability, by inducing action potentials, and promote the release of BDNF, enhancing long-term potentiation at synapses. This method has been shown to improve overall cognitive levels in AD patients [263]. Combining cognitive training with rTMS can further enhance memory in AD patients. Both transcranial direct current stimulation (tDCS) and rTMS have been demonstrated to increase striatal dopamine levels, improving executive function [264], working memory [265] and episodic memory [266] in PD patients.

These behavioral and neuromodulation interventions offer promising avenues for improving cognitive functions in individuals with neurodegenerative diseases, complementing pharmacological approaches and providing holistic treatment strategies.

## CONCLUSION

The wakefulness system regulates memory encoding and retrieval in an activity-dependent manner via the modulation of arousal levels. This system can directly influence memory-related brain regions by affecting memory engram cells, synaptic plasticity, neuronal oscillation and AHN, thereby regulating both memory encoding and retrieval. In aging, PND and neurodegenerative diseases such as AD and PD, impairment of the wakefulness system and reduced arousal levels are significant contributors to memory impairment. Pharmacological treatments, behavioral interventions and neuromodulation strategies aimed at enhancing arousal levels are emerging as promising approaches for treating memory deficits.

Although the focus of this review is on behavioral and circuitry mechanisms, molecular processes are integral to how wakefulness regulates memory. Wake-promoting neurons utilize neurotransmitters such as orexin, acetylcholine, dopamine, noradrenaline and serotonin to modulate hippocampal and cortical neuronal excitability, synaptic plasticity and oscillatory dynamics. These neuromodulators influence intracellular signaling pathways, including cAMP/PKA, Ca²⁺-dependent kinases and cAMP response element-binding protein (CREB)-mediated transcription, which are critical for LTP and the stabilization of memory engrams [267,268]. Moreover, wakefulness can affect neurotrophic factors, such as BDNF, and modulate synaptic protein synthesis, contributing to the formation and maintenance of synaptic connections underlying memory encoding and retrieval [269,270]. The molecular mechanisms of wakefulness regulation of memory are complex and remain largely beyond the scope of this review. Future studies integrating molecular, circuit and behavioral approaches will be essential to fully elucidate how wakefulness orchestrates memory at multiple levels and represents a critical avenue for future research, and could identify novel therapeutic targets for memory disorders.

As research progresses, additional wakefulness systems will be identified, their functions further clarified, and the mechanisms of wakefulness abnormalities inducing memory deficits elucidated. This will help uncover how wakefulness system impairments cause memory deficits in aging, neurodegenerative diseases and general anesthesia. Targeting the wakefulness system, especially those aspects that act on memory brain regions, can alleviate memory impairment, but precise control remains challenging. The development of targeted drugs and neural modulation strategies is also an important research direction.

## References

[1] Carvalho DZ, St Louis EK, Knopman DS et al. Association of excessive daytime sleepiness with longitudinal beta-amyloid accumulation in elderly persons without dementia. JAMA Neurol 2018; 75: 672–80. 10.1001/jamaneurol.2018.0049 29532057 PMC5885188

[2] Oh J, Eser RA, Ehrenberg AJ et al. Profound degeneration of wake-promoting neurons in Alzheimer’s disease. Alzheimers Dement 2019; 15: 1253–63. 10.1016/j.jalz.2019.06.3916 31416793 PMC6801040

[3] Janus C, Koperwas JS, Janus M et al. Rearing environment and radial maze exploration in mice. Behav Processes 1995; 34: 129–40. 10.1016/0376-6357(94)00060-t 24897513

[4] Scammell TE, Arrigoni E, Lipton JO. Neural circuitry of wakefulness and sleep. Neuron 2017; 93: 747–65. 10.1016/j.neuron.2017.01.014 28231463 PMC5325713

[5] Klinzing JG, Niethard N, Born J. Mechanisms of systems memory consolidation during sleep. Nat Neurosci 2019; 22: 1598–610. 10.1038/s41593-019-0467-3 31451802

[6] Diekelmann S, Born J. The memory function of sleep. Nat Rev Neurosci 2010; 11: 114–26. 10.1038/nrn2762 20046194

[7] Moruzzi G, Magoun HW. Brain stem reticular formation and activation of the EEG. Electroencephalogr Clin Neurophysiol 1949; 1: 455–73. 18421835

[8] Saper CB, Scammell TE, Lu J. Hypothalamic regulation of sleep and circadian rhythms. Nature 2005; 437: 1257–63. 10.1038/nature04284 16251950

[9] Sulaman BA, Wang Su, Tyan J et al. Neuro-orchestration of sleep and wakefulness. Nat Neurosci 2023; 26: 196–212. 10.1038/s41593-022-01236-w 36581730 PMC12714371

[10] Qiu MH, Chen MC, Fuller PM et al. Stimulation of the pontine parabrachial nucleus promotes wakefulness via extra-thalamic forebrain circuit nodes. Curr Biol 2016; 26: 2301–12. 10.1016/j.cub.2016.07.054 27546576 PMC5025760

[11] Berridge CW, Schmeichel BE, España RA. Noradrenergic modulation of wakefulness/arousal. Sleep Med Rev 2012; 16: 187–97. 10.1016/j.smrv.2011.12.003 22296742 PMC3278579

[12] Ren S, Wang Y, Yue F et al. The paraventricular thalamus is a critical thalamic area for wakefulness. Science 2018; 362: 429–34. 10.1126/science.aat2512 30361367

[13] Pedersen NP, Ferrari L, Venner A et al. Supramammillary glutamate neurons are a key node of the arousal system. Nat Commun 2017; 8: 1405. 10.1038/s41467-017-01004-6 29123082 PMC5680228

[14] Sun Li, Liu R, Guo F et al. Parabrachial nucleus circuit governs neuropathic pain-like behavior. Nat Commun 2020; 11: 5974. 10.1038/s41467-020-19767-w 33239627 PMC7688648

[15] Yang WZ, Xie H, Du X et al. A parabrachial-hypothalamic parallel circuit governs cold defense in mice. Nat Commun 2023; 14: 4924. 10.1038/s41467-023-40504-6 37582782 PMC10427655

[16] Tokita K, Boughter JD Jr. Topographic organizations of taste-responsive neurons in the parabrachial nucleus of C57BL/6J mice: An electrophysiological mapping study. Neuroscience 2016; 316: 151–66. 10.1016/j.neuroscience.2015.12.030 26708748 PMC4724509

[17] Chen J, Gannot N, Li X et al. Control of emotion and wakefulness by neurotensinergic neurons in the parabrachial nucleus. Neurosci Bull 2023; 39: 589–601. 10.1007/s12264-022-00994-8 36522525 PMC10073397

[18] Zitnik GA, Curtis AL, Wood SK et al. Adolescent social stress produces an enduring activation of the rat locus coeruleus and alters its coherence with the prefrontal cortex. Neuropsychopharmacology 2016; 41: 1376–85. 10.1038/npp.2015.289 26361057 PMC4793122

[19] Gao C, Gohel CA, Leng Y et al. Molecular and spatial profiling of the paraventricular nucleus of the thalamus. eLife 2023; 12: e81818. 10.7554/eLife.81818 36867023 PMC10014079

[20] Zhu Y, Nachtrab G, Keyes PC et al. Dynamic salience processing in paraventricular thalamus gates associative learning. Science 2018; 362: 423–9. 10.1126/science.aat0481 30361366 PMC6521722

[21] Li Y, Bao H, Luo Y et al. Supramammillary nucleus synchronizes with dentate gyrus to regulate spatial memory retrieval through glutamate release. eLife 2020; 9: e53129. 10.7554/eLife.53129 32167473 PMC7069722

[22] Luo Y-J, Li L, Chen Ze-Ka et al. Segregated supramammillary-dentate gyrus circuits modulate cognitive and affective function in healthy and Alzheimer’s disease model mice. Neuron 2025; S0896-6273(25)00672-5. 10.1016/j.neuron.2025.09.006 PMC12581905 41045929

[23] Gielow MR, Zaborszky L. The input-output relationship of the cholinergic basal forebrain. Cell Rep 2017; 18: 1817–30. 10.1016/j.celrep.2017.01.060 28199851 PMC5725195

[24] Dong H, Chen Ze-Ka, Guo H et al. Striatal neurons expressing dopamine D(1) receptor promote wakefulness in mice. Curr Biol 2022; 32: 600–13. 10.1016/j.cub.2021.12.026 35021048

[25] Naganuma F, Nakamura T, Kuroyanagi H et al. Chemogenetic modulation of histaminergic neurons in the tuberomamillary nucleus alters territorial aggression and wakefulness. Sci Rep 2021; 11: 17935. 10.1038/s41598-021-95497-3 34504120 PMC8429727

[26] Mohebi A, Pettibone JR, Hamid AA et al. Dissociable dopamine dynamics for learning and motivation. Nature 2019; 570: 65–70. 10.1038/s41586-019-1235-y 31118513 PMC6555489

[27] Yoo JiH, Zell V, Gutierrez-Reed N et al. Ventral tegmental area glutamate neurons co-release GABA and promote positive reinforcement. Nat Commun 2016; 7: 13697. 10.1038/ncomms13697 27976722 PMC5171775

[28] Li Ya-D, Luo Y-J, Xu W et al. Ventral pallidal GABAergic neurons control wakefulness associated with motivation through the ventral tegmental pathway. Mol Psychiatry 2021; 26: 2912–28. 10.1038/s41380-020-00906-0 33057171 PMC8505244

[29] Luo Y-J, Li Ya-D, Wang Lu et al. Nucleus accumbens controls wakefulness by a subpopulation of neurons expressing dopamine D(1) receptors. Nat Commun 2018; 9: 1576. 10.1038/s41467-018-03889-3 29679009 PMC5910424

[30] Yamashita T, Yamanaka A. Lateral hypothalamic circuits for sleep–wake control. Curr Opin Neurobiol 2017; 44: 94–100. 10.1016/j.conb.2017.03.020 28427008

[31] Herrera CG, Cadavieco MC, Jego S et al. Hypothalamic feedforward inhibition of thalamocortical network controls arousal and consciousness. Nat Neurosci 2016; 19: 290–8. 10.1038/nn.4209 26691833 PMC5818272

[32] Wang R‐F, Guo H, Jiang S‐Yu et al. Control of wakefulness by lateral hypothalamic glutamatergic neurons in male mice. J Neurosci Res 2021; 99: 1689–703. 10.1002/jnr.24828 33713502

[33] Teissier A, Chemiakine A, Inbar B et al. Activity of Raphé serotonergic neurons controls emotional behaviors. Cell Rep 2015; 13: 1965–76. 10.1016/j.celrep.2015.10.061 26655908 PMC4756479

[34] Liu Z, Zhou J, Li Yi et al. Dorsal raphe neurons signal reward through 5-HT and glutamate. Neuron 2014; 81: 1360–74. 10.1016/j.neuron.2014.02.010 24656254 PMC4411946

[35] Fonseca MS, Murakami M, Mainen ZF. Activation of dorsal raphe serotonergic neurons promotes waiting but is not reinforcing. Curr Biol 2015; 25: 306–15. 10.1016/j.cub.2014.12.002 25601545

[36] Cho JR, Chen X, Kahan A et al. Dorsal raphe dopamine neurons signal motivational salience dependent on internal state, expectation, and behavioral context. J Neurosci 2021; 41: 2645–55. 10.1523/jneurosci.2690-20.2021 33563725 PMC8018733

[37] Fukushi I, Yokota S, Okada Y. The role of the hypothalamus in modulation of respiration. Respir Physiol Neurobiol 2019; 265: 172–9. 10.1016/j.resp.2018.07.003 30009993

[38] Mińczuk K, Schlicker E, Malinowska B. Cross-talk between CB_1_, AT_1_, AT_2_ and Mas receptors responsible for blood pressure control in the paraventricular nucleus of hypothalamus in conscious spontaneously hypertensive rats and their normotensive controls. Cells 2022; 11: 1542. 10.3390/cells11091542 35563848 PMC9101384

[39] Ramchandra R, Hood SG, Frithiof R et al. The role of the paraventricular nucleus of the hypothalamus in the regulation of cardiac and renal sympathetic nerve activity in conscious normal and heart failure sheep. J Physiol 2013; 591: 93–107. 10.1113/jphysiol.2012.236059 22615431 PMC3630774

[40] Liu Q, Bell BJ, Kim DW et al. A clock-dependent brake for rhythmic arousal in the dorsomedial hypothalamus. Nat Commun 2023; 14: 6381. 10.1038/s41467-023-41877-4 37821426 PMC10567910

[41] Chou TC, Scammell TE, Gooley JJ et al. Critical role of dorsomedial hypothalamic nucleus in a wide range of behavioral circadian rhythms. J Neurosci 2003; 23: 10691–702. 10.1523/jneurosci.23-33-10691.2003 14627654 PMC6740926

[42] Pho H, Berger S, Freire C et al. Leptin receptor expression in the dorsomedial hypothalamus stimulates breathing during NREM sleep in db/db mice. Sleep 2021; 44: zsab046. 10.1093/sleep/zsab046 33624805 PMC8193564

[43] Do J, Chang Z, Sekerková G et al. A leptin-mediated neural mechanism linking breathing to metabolism. Cell Rep 2020; 33: 108358. 10.1016/j.celrep.2020.108358 33176139 PMC7698346

[44] Piñol RA et al. Brs3 neurons in the mouse dorsomedial hypothalamus regulate body temperature, energy expenditure, and heart rate, but not food intake. Nat Neurosci 2018; 21: 1530–40. 10.1038/s41593-018-0249-3 30349101 PMC6203600

[45] Yackle K, Schwarz LA, Kam K et al. Breathing control center neurons that promote arousal in mice. Science 2017; 355: 1411–5. 10.1126/science.aai7984 28360327 PMC5505554

[46] Toor RUlAS, Sun QJ, Kumar NN et al. Neurons in the intermediate reticular nucleus coordinate postinspiratory activity, swallowing, and respiratory-sympathetic coupling in the rat. J Neurosci 2019; 39: 9757–66. 10.1523/jneurosci.0502-19.2019 31666354 PMC6891060

[47] Ross Ca, Ruggiero Da, Park Dh et al. Tonic vasomotor control by the rostral ventrolateral medulla: Effect of electrical or chemical stimulation of the area containing C1 adrenaline neurons on arterial pressure, heart rate, and plasma catecholamines and vasopressin. J Neurosci 1984; 4: 474–94. 10.1523/jneurosci.04-02-00474.1984 6699683 PMC6564896

[48] McGinley MJ, Vinck M, Reimer J et al. Waking state: Rapid variations modulate neural and behavioral responses. Neuron 2015; 87: 1143–61. 10.1016/j.neuron.2015.09.012 26402600 PMC4718218

[49] Vallat R, Meunier D, Nicolas A et al. Hard to wake up? The cerebral correlates of sleep inertia assessed using combined behavioral, EEG and fMRI measures. Neuroimage 2019; 184: 266–78. 10.1016/j.neuroimage.2018.09.033 30223060

[50] Koch C, Massimini M, Boly M et al. Neural correlates of consciousness: Progress and problems. Nat Rev Neurosci 2016; 17: 307–21. 10.1038/nrn.2016.22 27094080

[51] Adamantidis AR, Gutierrez Herrera C, Gent TC. Oscillating circuitries in the sleeping brain. Nat Rev Neurosci 2019; 20: 746–62. 10.1038/s41583-019-0223-4 31616106

[52] Gervasoni D, Lin S-C, Ribeiro S et al. Global forebrain dynamics predict rat behavioral states and their transitions. J Neurosci 2004; 24: 11137–47. 10.1523/JNEUROSCI.3524-04.2004 15590930 PMC6730270

[53] Pivik RT, Harman K. A reconceptualization of EEG alpha activity as an index of arousal during sleep: All alpha activity is not equal. J Sleep Res 1995; 4: 131–7. 10.1111/j.1365-2869.1995.tb00161.x 10607151

[54] Wang YL, Avigdor T, Hannan S et al. Intracerebral dynamics of sleep arousals: A combined scalp-intracranial EEG study. J Neurosci 2024; 44: e0617232024. 10.1523/JNEUROSCI.0617-23.2024 38471781 PMC11026366

[55] Vyazovskiy VV, Olcese U, Hanlon EC et al. Local sleep in awake rats. Nature 2011; 472: 443–7. 10.1038/nature10009 21525926 PMC3085007

[56] Reimer J, Froudarakis E, Cadwell CR et al. Pupil fluctuations track fast switching of cortical states during quiet wakefulness. Neuron 2014; 84: 355–62. 10.1016/j.neuron.2014.09.033 25374359 PMC4323337

[57] Vinck M, Batista-Brito R, Knoblich U et al. Arousal and locomotion make distinct contributions to cortical activity patterns and visual encoding. Neuron 2015; 86: 740–54. 10.1016/j.neuron.2015.03.028 25892300 PMC4425590

[58] Liu J, Wei W, Kuang H et al. Heart rate and heart rate variability assessment identifies individual differences in fear response magnitudes to earthquake, free fall, and air puff in mice. PLoS One 2014; 9: e93270. 10.1371/journal.pone.0093270 24667366 PMC3965551

[59] Turner KL, Gheres KW, Drew PJ. Relating pupil diameter and blinking to cortical activity and hemodynamics across arousal states. J Neurosci 2023; 43: 949–64. 10.1523/JNEUROSCI.1244-22.2022 36517240 PMC9908322

[60] Ciria LF, Suárez-Pinilla M, Williams AG et al. Different underlying mechanisms for high and low arousal in probabilistic learning in humans. Cortex 2021; 143: 180–94. 10.1016/j.cortex.2021.07.002 34450566

[61] Kreibig SD . Autonomic nervous system activity in emotion: A review. Biol Psychol 2010; 84: 394–421. 10.1016/j.biopsycho.2010.03.010 20371374

[62] Hou TY, Cai WP. What emotion dimensions can affect working memory performance in healthy adults? A review. World J Clin Cases 2022; 10: 401–11. 10.12998/wjcc.v10.i2.401 35097065 PMC8771390

[63] Knight M, Mather M. Reconciling findings of emotion-induced memory enhancement and impairment of preceding items. Emotion 2009; 9: 763–81. 10.1037/a0017281 20001121 PMC2917000

[64] Zheng Y, Tao S, Liu Y et al. Basal forebrain-dorsal hippocampus cholinergic circuit regulates olfactory associative learning. Int J Mol Sci 2022; 23: 8472. 10.3390/ijms23158472 35955605 PMC9368792

[65] Boyce R, Glasgow SD, Williams S et al. Causal evidence for the role of REM sleep theta rhythm in contextual memory consolidation. Science 2016; 352: 812–6. 10.1126/science.aad5252 27174984

[66] Liao Y, Wen R, Fu S et al. Spatial memory requires hypocretins to elevate medial entorhinal gamma oscillations. Neuron 2024; 112: 155–73. 10.1016/j.neuron.2023.10.012 37944520

[67] Nagai Y, Kisaka Y, Nomura K et al. Dorsal raphe serotonergic neurons preferentially reactivate dorsal dentate gyrus cell ensembles associated with positive experience. Cell Rep 2023; 42: 112149. 10.1016/j.celrep.2023.112149 36821440

[68] Seo D-Oh, Zhang ET, Piantadosi SC et al. A locus coeruleus to dentate gyrus noradrenergic circuit modulates aversive contextual processing. Neuron 2021; 109: 2116–30. 10.1016/j.neuron.2021.05.006 34081911 PMC8754261

[69] Barnes TD, Kubota Y, Hu D et al. Activity of striatal neurons reflects dynamic encoding and recoding of procedural memories. Nature 2005; 437: 1158–61. 10.1038/nature04053 16237445

[70] Josselyn SA, Frankland PW. Memory allocation: Mechanisms and function. Annu Rev Neurosci 2018; 41: 389–413. 10.1146/annurev-neuro-080317-061956 29709212 PMC9623596

[71] Pignatelli M, Ryan TJ, Roy DS et al. Engram cell excitability state determines the efficacy of memory retrieval. Neuron 2019; 101: 274–84. 10.1016/j.neuron.2018.11.029 30551997

[72] Liu Xu, Ramirez S, Pang PT et al. Optogenetic stimulation of a hippocampal engram activates fear memory recall. Nature 2012; 484: 381–5. 10.1038/nature11028 22441246 PMC3331914

[73] Mozzachiodi R, Byrne JH. More than synaptic plasticity: Role of nonsynaptic plasticity in learning and memory. Trends Neurosci 2010; 33: 17–26. 10.1016/j.tins.2009.10.001 19889466 PMC2815214

[74] Mocle AJ, Ramsaran AI, Jacob AD et al. Excitability mediates allocation of pre-configured ensembles to a hippocampal engram supporting contextual conditioned threat in mice. Neuron 2024; 112: 1487–97. 10.1016/j.neuron.2024.02.007 38447576 PMC11065628

[75] He C, Luo F, Chen X et al. Superficial layer-specific histaminergic modulation of medial entorhinal cortex required for spatial learning. Cereb Cortex 2016; 26: 1590–608. 10.1093/cercor/bhu322 25595181 PMC6276960

[76] Alejandre-García T, Kim S, Pérez-Ortega J et al. Intrinsic excitability mechanisms of neuronal ensemble formation. eLife 2022; 11: e77470. 10.7554/eLife.77470 35506662 PMC9197391

[77] Carrillo-Reid L, Han S, Yang W et al. Controlling visually guided behavior by holographic recalling of cortical ensembles. Cell 2019; 178: 447–57. 10.1016/j.cell.2019.05.045 31257030 PMC6747687

[78] Lesuis SL, Park S, Hoorn A et al. Stress disrupts engram ensembles in lateral amygdala to generalize threat memory in mice. Cell 2025; 188: 121–40. 10.1016/j.cell.2024.10.034 39549697 PMC11726195

[79] Ramsaran AI, Wang Y, Golbabaei A et al. A shift in the mechanisms controlling hippocampal engram formation during brain maturation. Science 2023; 380: 543–51. 10.1126/science.ade6530 37141366

[80] Ryan TJ, Roy DS, Pignatelli M et al. Engram cells retain memory under retrograde amnesia. Science 2015; 348: 1007–13. 10.1126/science.aaa5542 26023136 PMC5583719

[81] Redondo RL, Kim J, Arons AL et al. Bidirectional switch of the valence associated with a hippocampal contextual memory engram. Nature 2014; 513: 426–30. 10.1038/nature13725 25162525 PMC4169316

[82] Zhou Y, Zhu H, Liu Z et al. A ventral CA1 to nucleus accumbens core engram circuit mediates conditioned place preference for cocaine. Nat Neurosci 2019; 22: 1986–99. 10.1038/s41593-019-0524-y 31719672

[83] Roy DS, Arons A, Mitchell TI et al. Memory retrieval by activating engram cells in mouse models of early Alzheimer’s disease. Nature 2016; 531: 508–12. 10.1038/nature17172 26982728 PMC4847731

[84] Okuyama T, Kitamura T, Roy DS et al. Ventral CA1 neurons store social memory. Science 2016; 353: 1536–41. 10.1126/science.aaf7003 27708103 PMC5493325

[85] Hsiang H, Epp JR, van den Oever MC et al. Manipulating a “cocaine engram” in mice. J Neurosci 2014; 34: 14115–27. 10.1523/JNEUROSCI.3327-14.2014 25319707 PMC6705287

[86] Yiu AP, Mercaldo V, Yan C et al. Neurons are recruited to a memory trace based on relative neuronal excitability immediately before training. Neuron 2014; 83: 722–35. 10.1016/j.neuron.2014.07.017 25102562

[87] Rashid AJ, Yan C, Mercaldo V et al. Competition between engrams influences fear memory formation and recall. Science 2016; 353: 383–7. 10.1126/science.aaf0594 27463673 PMC6737336

[88] Carrillo-Reid L, Yang W, Bando Y et al. Imprinting and recalling cortical ensembles. Science 2016; 353: 691–4. 10.1126/science.aaf7560 27516599 PMC5482530

[89] Davis CD, Jones FL, Derrick BE. Novel environments enhance the induction and maintenance of long-term potentiation in the dentate gyrus. J Neurosci 2004; 24: 6497–506. 10.1126/science.1239073 15269260 PMC6729872

[90] Brown TH, Chapman PF, Kairiss EW et al. Long-term synaptic potentiation. Science 1988; 242: 724–8. 10.1126/science.2903551 2903551

[91] Magee JC, Grienberger C. Synaptic plasticity forms and functions. Annu Rev Neurosci 2020; 43: 95–117. 10.1146/annurev-neuro-090919-022842 32075520

[92] Martin SJ, Grimwood PD, Morris RG. Synaptic plasticity and memory: An evaluation of the hypothesis. Annu Rev Neurosci 2000; 23: 649–711. 10.1146/annurev.neuro.23.1.649 10845078

[93] Villarreal DM, Do V, Haddad E et al. NMDA receptor antagonists sustain LTP and spatial memory: Active processes mediate LTP decay. Nat Neurosci 2002; 5: 48–52. 10.1038/nn776 11740500

[94] Rumpel S, LeDoux J, Zador A et al. Postsynaptic receptor trafficking underlying a form of associative learning. Science 2005; 308: 83–8. 10.1126/science.1103944 15746389

[95] Bushey D, Tononi G, Cirelli C. Sleep and synaptic homeostasis: Structural evidence in *Drosophila*. Science 2011; 332: 1576–81. 10.1126/science.1202839 21700878 PMC3128387

[96] Chandra R, Farah F, Muñoz-Lobato F et al. Sleep is required to consolidate odor memory and remodel olfactory synapses. Cell 2023; 186: 2911–28. 10.1016/j.cell.2023.05.006 37269832 PMC10354834

[97] Suppermpool A, Lyons DG, Broom E et al. Sleep pressure modulates single-neuron synapse number in zebrafish. Nature 2024; 629: 639–45. 10.1038/s41586-024-07367-3 38693264 PMC11096099

[98] de Vivo L, Bellesi M, Marshall W et al. Ultrastructural evidence for synaptic scaling across the wake/sleep cycle. Science 2017; 355: 507–10. 10.1126/science.aah5982 28154076 PMC5313037

[99] Spano GM, Banningh SW, Marshall W et al. Sleep deprivation by exposure to novel objects increases synapse density and axon-spine interface in the hippocampal CA1 region of adolescent mice. J Neurosci 2019; 39: 6613–25. 10.1523/JNEUROSCI.0380-19.2019 31263066 PMC6703893

[100] Maret S, Faraguna U, Nelson AB et al. Sleep and waking modulate spine turnover in the adolescent mouse cortex. Nat Neurosci 2011; 14: 1418–20. 10.1038/nn.2934 21983682 PMC3203346

[101] Yang G, Lai CSW, Cichon J et al. Sleep promotes branch-specific formation of dendritic spines after learning. Science 2014; 344: 1173–8. 10.1126/science.1249098 24904169 PMC4447313

[102] Li W, Ma L, Yang G et al. REM sleep selectively prunes and maintains new synapses in development and learning. Nat Neurosci 2017; 20: 427–37. 10.1038/nn.4479 28092659 PMC5535798

[103] Rodenkirch C, Liu Y, Schriver BJ et al. Locus coeruleus activation enhances thalamic feature selectivity via norepinephrine regulation of intrathalamic circuit dynamics. Nat Neurosci 2019; 22: 120–33. 10.1038/s41593-018-0283-1 30559472 PMC6301066

[104] Dasgupta A, Baby N, Krishna K et al. Substance P induces plasticity and synaptic tagging/capture in rat hippocampal area CA2. Proc Natl Acad Sci USA 2017; 114: E8741–9. 10.1073/pnas.1711267114 28973908 PMC5642719

[105] Buzsáki G, Anastassiou CA, Koch C. The origin of extracellular fields and currents–EEG, ECoG, LFP and spikes. Nat Rev Neurosci 2012; 13: 407–20. 10.1038/nrn3241 22595786 PMC4907333

[106] Vertes RP, Kocsis B. Brainstem-diencephalo-septohippocampal systems controlling the theta rhythm of the hippocampus. Neuroscience 1997; 81: 893–926. 10.1016/s0306-4522(97)00239-x 9330355

[107] Dai JX, Han HL, Tian M et al. Enhanced contextual fear memory in central serotonin-deficient mice. Proc Natl Acad Sci USA 2008; 105: 11981–6. 10.1073/pnas.0801329105 18695238 PMC2575315

[108] Ohmura Yu, Izumi T, Yamaguchi T et al. The serotonergic projection from the median raphe nucleus to the ventral hippocampus is involved in the retrieval of fear memory through the corticotropin-releasing factor type 2 receptor. Neuropsychopharmacology 2010; 35: 1271–8. 10.1038/npp.2009.229 20072117 PMC3055345

[109] Kocsis B, Varga V, Dahan L et al. Serotonergic neuron diversity: Identification of raphe neurons with discharges time-locked to the hippocampal theta rhythm. Proc Natl Acad Sci USA 2006; 103: 1059–64. 10.1073/pnas.0508360103 16418294 PMC1347988

[110] Murchison CF, Zhang X-Y, Zhang W-P et al. A distinct role for norepinephrine in memory retrieval. Cell 2004; 117: 131–43. 10.1016/s0092-8674(04)00259-4 15066288

[111] Vassalli A, Franken P. Hypocretin (orexin) is critical in sustaining theta/gamma-rich waking behaviors that drive sleep need. Proc Natl Acad Sci USA 2017; 114: E5464–73. 10.1073/pnas.1700983114 28630298 PMC5502606

[112] Anaclet C, Pedersen NP, Ferrari LL et al. Basal forebrain control of wakefulness and cortical rhythms. Nat Commun 2015; 6: 8744. 10.1038/ncomms9744 26524973 PMC4659943

[113] Chen L, Yin D, Wang TX et al. Basal forebrain cholinergic neurons primarily contribute to inhibition of electroencephalogram delta activity, rather than inducing behavioral wakefulness in mice. Neuropsychopharmacology 2016; 41: 2133–46. 10.1038/npp.2016.13 26797244 PMC4908644

[114] Kaur S, Junek A, Black MA et al. Effects of ibotenate and 192IgG-saporin lesions of the nucleus basalis magnocellularis/substantia innominata on spontaneous sleep and wake states and on recovery sleep after sleep deprivation in rats. J Neurosci 2008; 28: 491–504. 10.1523/JNEUROSCI.1585-07.2008 18184792 PMC6670515

[115] Conner JM, Culberson A, Packowski C et al. Lesions of the basal forebrain cholinergic system impair task acquisition and abolish cortical plasticity associated with motor skill learning. Neuron 2003; 38: 819–29. 10.1016/s0896-6273(03)00288-5 12797965

[116] Everitt BJ, Robbins TW. Central cholinergic systems and cognition. Annu Rev Psychol 1997; 48: 649–84. 10.1146/annurev.psych.48.1.649 9046571

[117] Ballinger EC, Ananth M, Talmage DA et al. Basal forebrain cholinergic circuits and signaling in cognition and cognitive decline. Neuron 2016; 91: 1199–218. 10.1016/j.neuron.2016.09.006 27657448 PMC5036520

[118] Tang FRu, Loke WK, Wong P et al. Radioprotective effect of ursolic acid in radiation-induced impairment of neurogenesis, learning and memory in adolescent BALB/c mouse. Physiol Behav 2017; 175: 37–46. 10.1016/j.physbeh.2017.03.027 28341234

[119] Luo Y, Coskun V, Liang A et al. Single-cell transcriptome analyses reveal signals to activate dormant neural stem cells. Cell 2015; 161: 1175–86. 10.1016/j.cell.2015.04.001 26000486 PMC4851109

[120] Sahay A, Scobie KN, Hill AS et al. Increasing adult hippocampal neurogenesis is sufficient to improve pattern separation. Nature 2011; 472: 466–70. 10.1038/nature09817 21460835 PMC3084370

[121] Berdugo‐Vega G, Lee C‐C, Garthe A et al. Adult-born neurons promote cognitive flexibility by improving memory precision and indexing. Hippocampus 2021; 31: 1068–79. 10.1002/hipo.23373 34174010

[122] Besnard A, Sahay A. Enhancing adult neurogenesis promotes contextual fear memory discrimination and activation of hippocampal-dorsolateral septal circuits. Behav Brain Res 2021; 399: 112917. 10.1016/j.bbr.2020.112917 32949641 PMC7855356

[123] Li YD, Luo YJ, Chen ZK et al. Hypothalamic modulation of adult hippocampal neurogenesis in mice confers activity-dependent regulation of memory and anxiety-like behavior. Nat Neurosci 2022; 25: 630–45. 10.1038/s41593-022-01065-x 35524139 PMC9287980

[124] Kumar D, Koyanagi I, Carrier-Ruiz A et al. Sparse activity of hippocampal adult-born neurons during REM sleep is necessary for memory consolidation. Neuron 2020; 107: 552–65. 10.1016/j.neuron.2020.05.008 32502462

[125] Gu Y, Arruda-Carvalho M, Wang J et al. Optical controlling reveals time-dependent roles for adult-born dentate granule cells. Nat Neurosci 2012; 15: 1700–6. 10.1038/nn.3260 23143513 PMC3509272

[126] Danielson NB, Kaifosh P, Zaremba JD et al. Distinct contribution of adult-born hippocampal granule cells to context encoding. Neuron 2016; 90: 101–12. 10.1016/j.neuron.2016.02.019 26971949 PMC4962695

[127] Zhou M, Li W, Huang S et al. mTOR inhibition ameliorates cognitive and affective deficits caused by Disc1 knockdown in adult-born dentate granule neurons. Neuron 2013; 77: 647–54. 10.1016/j.neuron.2012.12.033 23439118 PMC3586374

[128] Zhu Y, Armstrong JN, Contractor A. Kainate receptors regulate the functional properties of young adult-born dentate granule cells. Cell Rep 2021; 36: 109751. 10.1016/j.celrep.2021.109751 34551304 PMC8525187

[129] Li YD, Luo YJ, Xie L et al. Activation of hypothalamic-enhanced adult-born neurons restores cognitive and affective function in Alzheimer’s disease. Cell stem cell 2023; 30: 415–32. 10.1016/j.stem.2023.02.006 37028406 PMC10150940

[130] Li YD, Luo YJ, Song J. Optimizing memory performance and emotional states: Multi-level enhancement of adult hippocampal neurogenesis. Curr Opin Neurobiol 2023; 79: 102693. 10.1016/j.conb.2023.102693 36822141 PMC10023407

[131] Forte N, Boccella S, Tunisi L et al. Orexin-A and endocannabinoids are involved in obesity-associated alteration of hippocampal neurogenesis, plasticity, and episodic memory in mice. Nat Commun 2021; 12: 6137. 10.1038/s41467-021-26388-4 34675233 PMC8531398

[132] Gulino R, Nunziata D, de Leo G et al. Hippocampal noradrenaline is a positive regulator of spatial working memory and neurogenesis in the rat. Int J Mol Sci 2023; 24: 5613. 10.3390/ijms24065613 36982688 PMC10052298

[133] Gould E . Serotonin and hippocampal neurogenesis. Neuropsychopharmacology 1999; 21: 46S–51S. 10.1016/S0893-133X(99)00045-7 10432488

[134] Zhao C, Deng W, Gage FH Mechanisms and functional implications of adult neurogenesis. Cell 2008; 132: 645–60. 10.1097/ALN.0b013e3181ca33a1 18295581

[135] Vutskits L, Xie Z. Lasting impact of general anaesthesia on the brain: Mechanisms and relevance. Nat Rev Neurosci 2016; 17: 705–17. 10.1038/nrn.2016.128 27752068

[136] Guo LY, Kaustov L, Brenna CTA et al. Cognitive deficits after general anaesthesia in animal models: A scoping review. Br J Anaesth 2023; 130: e351–60. 10.1016/j.bja.2022.10.004 36402576

[137] Zelmann R, Paulk AC, Tian F et al. Differential cortical network engagement during states of un/consciousness in humans. Neuron 2023; 111: 3479–95. 10.1016/j.neuron.2023.08.007 37659409 PMC10843836

[138] Mashour GA, Palanca BJA, Basner M et al. Recovery of consciousness and cognition after general anesthesia in humans. eLife 2021; 10: 59525. 10.7554/eLife.59525 PMC8163502 33970101

[139] Zurek AA, Yu J, Wang D-S et al. Sustained increase in α5GABA_A_ receptor function impairs memory after anesthesia. J Clin Invest 2014; 124: 5437–41. 10.1172/JCI76669 25365226 PMC4348961

[140] Nir T, Raizman R, Meningher I et al. Lateralisation of subcortical functional connectivity during and after general anaesthesia. Br J Anaesth 2022; 128: 65–76. 10.1016/j.bja.2021.08.033 34802696 PMC8787782

[141] Hu J-J, Liu Y, Yao H et al. Emergence of consciousness from anesthesia through ubiquitin degradation of KCC2 in the ventral posteromedial nucleus of the thalamus. Nat Neurosci 2023; 26: 751–64. 10.1038/s41593-023-01290-y 36973513

[142] Zhu XN, Li J, Qiu GL et al. Propofol exerts anti-anhedonia effects via inhibiting the dopamine transporter. Neuron 2023; 111: 1626–36. 10.1016/j.neuron.2023.02.017 36917979

[143] Jevtovic-Todorovic V, Hartman RE, Izumi Y et al. Early exposure to common anesthetic agents causes widespread neurodegeneration in the developing rat brain and persistent learning deficits. J Neurosci 2003; 23: 876–82. 10.1523/JNEUROSCI.23-03-00876.2003 12574416 PMC6741934

[144] Yang W, Chini M, Pöpplau JA et al. Anesthetics fragment hippocampal network activity, alter spine dynamics, and affect memory consolidation. PLoS Biol 2021; 19: e3001146. 10.1371/journal.pbio.3001146 33793545 PMC8016109

[145] Cai P, Su W-K, Zhang J-S et al. Facilitation of behavioral and cortical emergence from isoflurane anesthesia by GABAergic neurons in basal forebrain. J Neurosci 2023; 43: 2907–20. 10.1523/JNEUROSCI.0628-22.2023 36868854 PMC10124952

[146] Haubrich J, Bernabo M, Nader K. Noradrenergic projections from the locus coeruleus to the amygdala constrain fear memory reconsolidation. eLife 2020; 9: e57010. 10.7554/eLife.57010 32420872 PMC7297527

[147] Chowdhury A, Luchetti A, Fernandes G et al. A locus coeruleus-dorsal CA1 dopaminergic circuit modulates memory linking. Neuron 2022; 110: 3374–88. 10.1016/j.neuron.2022.08.001 36041433 PMC10508214

[148] Fu B, Yu T, Yuan J et al. Noradrenergic transmission in the central medial thalamic nucleus modulates the electroencephalographic activity and emergence from propofol anesthesia in rats. J Neurochem 2017; 140: 862–73. 10.1111/jnc.13939 28092095

[149] Taylor NE, Van Dort CJ, Kenny JD et al. Optogenetic activation of dopamine neurons in the ventral tegmental area induces reanimation from general anesthesia. Proc Natl Acad Sci USA 2016; 113: 12826–31. 10.1073/pnas.1614340113 27791160 PMC5111696

[150] Sayegh FJP, Mouledous L, Macri C et al. Ventral tegmental area dopamine projections to the hippocampus trigger long-term potentiation and contextual learning. Nat Commun 2024; 15: 4100. 10.1038/s41467-024-47481-4 38773091 PMC11109191

[151] Xin J, Shan W, Li J et al. Activation of the lateral habenula-ventral tegmental area neural circuit contributes to postoperative cognitive dysfunction in mice. Adv Sci (Weinh) 2022; 9: e2202228. 10.1002/advs.202202228 35616407 PMC9353455

[152] Yang L, Zou B, Xiong X et al. Hypocretin/orexin neurons contribute to hippocampus-dependent social memory and synaptic plasticity in mice. J Neurosci 2013; 33: 5275–84. 10.1523/JNEUROSCI.3200-12.2013 23516292 PMC3640412

[153] Mavanji V, Butterick TA, Duffy CM et al. Orexin/hypocretin treatment restores hippocampal-dependent memory in orexin-deficient mice. Neurobiol Learn Mem 2017; 146: 21–30. 10.1016/j.nlm.2017.10.014 29107703 PMC5798856

[154] Zhang LN, Li ZJ, Tong L et al. Orexin-A facilitates emergence from propofol anesthesia in the rat. Anesth Analg 2012; 115: 789–96. 10.1213/ANE.0b013e3182645ea3 22798527

[155] Zhao S, Wang S, Li H et al. Activation of orexinergic neurons inhibits the anesthetic effect of desflurane on consciousness state via paraventricular thalamic nucleus in rats. Anesth Analg 2021; 133: 781–93. 10.1213/ANE.0000000000005651 34403389

[156] Zhou W, Cheung K, Kyu S et al. Activation of orexin system facilitates anesthesia emergence and pain control. Proc Natl Acad Sci USA 2018; 115: E10740–7. 10.1073/pnas.1808622115 30348769 PMC6233126

[157] Sengupta A, Holmes A. A discrete dorsal raphe to basal amygdala 5-HT circuit calibrates aversive memory. Neuron 2019; 103: 489–505 e487. 10.1016/j.neuron.2019.05.029 31204082 PMC6687558

[158] Li Ao, Li R, Ouyang P et al. Dorsal raphe serotonergic neurons promote arousal from isoflurane anesthesia. CNS Neurosci Ther 2021; 27: 941–50. 10.1111/cns.13656 33973716 PMC8265942

[159] Wang YL, Wang L, Xu W et al. Paraventricular thalamus controls consciousness transitions during propofol anaesthesia in mice. Br J Anaesth 2023; 130: 698–708. 10.1016/j.bja.2023.01.016 36828739

[160] Ito M, Nagase M, Tohyama S et al. The parabrachial-to-amygdala pathway provides aversive information to induce avoidance behavior in mice. Mol Brain 2021; 14: 94. 10.1186/s13041-021-00807-5 34167570 PMC8223383

[161] Wang TX, Xiong B, Xu W et al. Activation of parabrachial nucleus glutamatergic neurons accelerates reanimation from sevoflurane anesthesia in mice. Anesthesiology 2019; 130: 106–18. 10.1097/ALN.0000000000002475 30325744

[162] Luo T-Y, Cai S, Qin Z-X et al. Basal forebrain cholinergic activity modulates isoflurane and propofol anesthesia. Front Neurosci 2020; 14: 559077. 10.3389/fnins.2020.559077 33192246 PMC7652994

[163] Chen L, Yang Z-L, Cheng J et al. Propofol decreases the excitability of cholinergic neurons in mouse basal forebrain via GABA_A_ receptors. Acta Pharmacol Sin 2019; 40: 755–61. 10.1038/s41401-018-0168-6 30367153 PMC6786414

[164] Liu W, Li J, Yang M et al. Chemical genetic activation of the cholinergic basal forebrain hippocampal circuit rescues memory loss in Alzheimer’s disease. Alzheimers Res Ther 2022; 14: 53. 10.1186/s13195-022-00994-w 35418161 PMC9006585

[165] Knox D, Keller SM. Cholinergic neuronal lesions in the medial septum and vertical limb of the diagonal bands of Broca induce contextual fear memory generalization and impair acquisition of fear extinction. Hippocampus 2016; 26: 718–26. 10.1002/hipo.22553 26606423 PMC5496651

[166] Lozano-Montes L, Dimanico M, Mazloum R et al. Optogenetic stimulation of basal forebrain parvalbumin neurons activates the default mode network and associated behaviors. Cell Rep 2020; 33: 108359. 10.1016/j.celrep.2020.108359 33176133

[167] Li Y, Chen L, Zhu D et al. Propofol downregulates the activity of glutamatergic neurons in the basal forebrain via affecting intrinsic membrane properties and postsynaptic GABA_A_Rs. Neuroreport 2020; 31: 1242–8. 10.1097/WNR.0000000000001540 33075002

[168] Tai SK, Ma J, Leung LS. Medial septal cholinergic neurons modulate isoflurane anesthesia. Anesthesiology 2014; 120: 392–402. 10.1097/ALN.0b013e3182a7cab6 23969562

[169] Leung LS, Ma J, Shen B et al. Medial septal lesion enhances general anesthesia response. Exp Neurol 2013; 247: 419–28. 10.1016/j.expneurol.2013.01.010 23376225

[170] Jin T, Chen R, Shao M et al. Dorsal hippocampus- and ACC-projecting medial septum neurons differentially contribute to the recollection of episodic-like memory. FASEB J 2020; 34: 11741–53. 10.1096/fj.202000398R 32652689

[171] Wang D, Guo Q, Zhou Y et al. GABAergic neurons in the dorsal–intermediate lateral septum regulate sleep–wakefulness and anesthesia in mice. Anesthesiology 2021; 135: 463–81. 10.1097/ALN.0000000000003868 34259824

[172] Li M, Li W, Liang S et al. BNST GABAergic neurons modulate wakefulness over sleep and anesthesia. Commun Biol 2024; 7: 339. 10.1038/s42003-024-06028-5 38503808 PMC10950862

[173] Bruzsik B, Biro L, Zelena D et al. Somatostatin neurons of the bed nucleus of stria terminalis enhance associative fear memory consolidation in mice. J Neurosci 2021; 41: 1982–95. 10.1523/JNEUROSCI.1944-20.2020 33468566 PMC7939080

[174] Zhao XP, Zhong F, Luo RY et al. Early-life sevoflurane exposure impairs fear memory by suppressing extracellular signal-regulated kinase signaling in the bed nucleus of stria terminalis GABAergic neurons. Neuropharmacology 2021; 191: 108584. 10.1016/j.neuropharm.2021.108584 33933475

[175] Bao W-W, Xu W, Pan G-J et al. Nucleus accumbens neurons expressing dopamine D1 receptors modulate states of consciousness in sevoflurane anesthesia. Curr Biol 2021; 31: 1893–902. 10.1016/j.cub.2021.02.011 33705720

[176] Wang L, Shen M, Yu Y et al. Optogenetic activation of GABAergic neurons in the nucleus accumbens decreases the activity of the ventral pallidum and the expression of cocaine-context-associated memory. Int J Neuropsychopharmacol 2014; 17: 753–63. 10.1017/S1461145713001570 24456857

[177] Zhou Y, Dong W, Qiu Y-K et al. Regulating the activity of GABAergic neurons in the ventral pallidum alters the general anesthesia effect of propofol. Neuropharmacology 2024; 257: 110032. 10.1016/j.neuropharm.2024.110032 38852839

[178] Akmese C, Sevinc C, Halim S et al. Differential role of GABAergic and cholinergic ventral pallidal neurons in behavioral despair, conditioned fear memory and active coping. Prog Neuropsychopharmacol Biol Psychiatry 2023; 125: 110760. 10.1016/j.pnpbp.2023.110760 37031946

[179] Li J-Y, Gao S-J, Li R-R et al. A neural circuit from the paraventricular thalamus to the bed nucleus of the stria terminalis for the regulation of states of consciousness during sevoflurane anesthesia in mice. Anesthesiology 2022; 136: 709–31. 10.1097/ALN.0000000000004195 35263424

[180] Ma J, du Hoffmann J, Kindel M et al. Divergent projections of the paraventricular nucleus of the thalamus mediate the selection of passive and active defensive behaviors. Nat Neurosci 2021; 24: 1429–40. 10.1038/s41593-021-00912-7 34413514 PMC8484052

[181] Redinbaugh MJ, Phillips JM, Kambi NA et al. Thalamus modulates consciousness via layer-specific control of cortex. Neuron 2020; 106: 66–75. 10.1016/j.neuron.2020.01.005 32053769 PMC7243351

[182] Luo T, Leung LS. Involvement of tuberomamillary histaminergic neurons in isoflurane anesthesia. Anesthesiology 2011; 115: 36–43. 10.1097/ALN.0b013e3182207655 21562401

[183] Costa A, Ducourneau E, Curti L et al. Chemogenetic activation or inhibition of histaminergic neurons bidirectionally modulates recognition memory formation and retrieval in male and female mice. Sci Rep 2024; 14: 11283. 10.1038/s41598-024-61998-0 38760416 PMC11101472

[184] Liu J, Liu X, Zhou WY et al. The activation of GABAergic neurons in the hypothalamic tuberomammillary nucleus attenuates sevoflurane and propofol-induced anesthesia in mice. Front Pharmacol 2023; 14: 1153735. 10.3389/fphar.2023.1153735 37426823 PMC10325722

[185] Yin J, Qin J, Lin Z et al. Glutamatergic neurons in the paraventricular hypothalamic nucleus regulate isoflurane anesthesia in mice. FASEB J 2023; 37: e22762. 10.1096/fj.202200974RR 36719765

[186] Zhao S, Li R, Li H et al. Lateral hypothalamic area glutamatergic neurons and their projections to the lateral habenula modulate the anesthetic potency of isoflurane in mice. Neurosci Bull 2021; 37: 934–46. 10.1007/s12264-021-00674-z 33847915 PMC8275739

[187] Sheng H, Lei C, Yuan Y et al. Nucleus accumbens circuit disinhibits lateral hypothalamus glutamatergic neurons contributing to morphine withdrawal memory in male mice. Nat Commun 2023; 14: 71. 10.1038/s41467-022-35758-5 36604429 PMC9814415

[188] Vazey EM, Aston-Jones G. Designer receptor manipulations reveal a role of the locus coeruleus noradrenergic system in isoflurane general anesthesia. Proc Natl Acad Sci USA 2014; 111: 3859–64. 10.1073/pnas.1310025111 24567395 PMC3956184

[189] Du W-J, Zhang R-W, Li J et al. The locus coeruleus modulates intravenous general anesthesia of zebrafish via a cooperative mechanism. Cell Rep 2018; 24: 3146–55. 10.1016/j.celrep.2018.08.046 30231998

[190] Ma H, Gu L, Wang Y et al. The states of different 5-HT receptors located in the dorsal raphe nucleus are crucial for regulating the awakening during general anesthesia. Mol Neurobiol 2023; 60: 6931–48. 10.1007/s12035-023-03519-0 37516665

[191] Gui H, Liu C, He H et al. Dopaminergic projections from the ventral tegmental area to the nucleus accumbens modulate sevoflurane anesthesia in mice. Front Cell Neurosci 2021; 15: 671473. 10.3389/fncel.2021.671473 33994950 PMC8119636

[192] Zhou X, Wang Y, Zhang C et al. The role of dopaminergic VTA neurons in general anesthesia. PLoS One 2015; 10: e0138187. 10.1371/journal.pone.0138187 26398236 PMC4580504

[193] Li J, Yu T, Shi F et al. Involvement of ventral periaqueductal gray dopaminergic neurons in propofol anesthesia. Neurochem Res 2018; 43: 838–47. 10.1007/s11064-018-2486-y 29417470

[194] Liu C, Zhou X, Zhu Q et al. Dopamine neurons in the ventral periaqueductal gray modulate isoflurane anesthesia in rats. CNS Neurosci Ther 2020; 26: 1121–33. 10.1111/cns.13447 32881314 PMC7564192

[195] Kim EJ, Horovitz O, Pellman BA et al. Dorsal periaqueductal gray-amygdala pathway conveys both innate and learned fear responses in rats. Proc Natl Acad Sci USA 2013; 110: 14795–800. 10.1073/pnas.1310845110 23959880 PMC3767534

[196] Li J, Hu R, Tan W et al. Activation of glutamatergic neurones in the pedunculopontine tegmental nucleus promotes cortical activation and behavioural emergence from sevoflurane-induced unconsciousness in mice. Br J Anaesth 2024; 132: 320–33. 10.1016/j.bja.2023.08.033 37953203

[197] Luo T, Yu S, Cai S et al. Parabrachial neurons promote behavior and electroencephalographic arousal from general anesthesia. Front Mol Neurosci 2018; 11: 420. 10.3389/fnmol.2018.00420 30564094 PMC6288364

[198] McDougall SJ, Bailey TW, Mendelowitz D et al. Propofol enhances both tonic and phasic inhibitory currents in second-order neurons of the solitary tract nucleus (NTS). Neuropharmacology 2008; 54: 552–63. 10.1016/j.neuropharm.2007.11.001 18082229 PMC2351956

[199] Liu C, Liu J, Zhou L et al. Lateral habenula glutamatergic neurons modulate isoflurane anesthesia in mice. Front Mol Neurosci 2021; 14: 628996. 10.3389/fnmol.2021.628996 33746711 PMC7969819

[200] Gelegen C, Miracca G, Ran MZ et al. Excitatory pathways from the lateral habenula enable propofol-induced sedation. Curr Biol 2018; 28: 580–7. 10.1016/j.cub.2017.12.050 29398217 PMC5835141

[201] Goutagny R, Loureiro M, Jackson J et al. Interactions between the lateral habenula and the hippocampus: Implication for spatial memory processes. Neuropsychopharmacology 2013; 38: 2418–26. 10.1038/npp.2013.142 23736315 PMC3799061

[202] Zhong H, Tong Li, Gu N et al. Endocannabinoid signaling in hypothalamic circuits regulates arousal from general anesthesia in mice. J Clin Invest 2017; 127: 2295–309. 10.1172/JCI91038 28463228 PMC5451249

[203] Silva BA, Mattucci C, Krzywkowski P et al. The ventromedial hypothalamus mediates predator fear memory. Eur J Neurosci 2016; 43: 1431–9. 10.1111/ejn.13239 26991018 PMC4899089

[204] Yang B, Ao Y, Liu Y et al. Activation of dopamine signals in the olfactory tubercle facilitates emergence from isoflurane anesthesia in mice. Neurochem Res 2021; 46: 1487–501. 10.1007/s11064-021-03291-4 33710536

[205] Martiros N, Kapoor V, Kim SE et al. Distinct representation of cue-outcome association by D1 and D2 neurons in the ventral striatum’s olfactory tubercle. eLife 2022; 11: e75463. 10.7554/eLife.75463 35708179 PMC9203051

[206] Sun XY, Liu L, Song YT et al. Two parallel medial prefrontal cortex-amygdala pathways mediate memory deficits via glutamatergic projection in surgery mice. Cell Rep 2023; 42: 112719. 10.1016/j.celrep.2023.112719 37392387

[207] Benn A, Barker GRI, Stuart SA et al. Optogenetic stimulation of prefrontal glutamatergic neurons enhances recognition memory. J Neurosci 2016; 36: 4930–9. 10.1523/JNEUROSCI.2933-15.2016 27147648 PMC4854963

[208] Zhang T, Yanagida J, Kamii H et al. Glutamatergic neurons in the medial prefrontal cortex mediate the formation and retrieval of cocaine-associated memories in mice. Addict Biol 2020; 25: e12723. 10.1111/adb.12723 30734456

[209] Pal D, Dean JG, Liu T et al. Differential role of prefrontal and parietal cortices in controlling level of consciousness. Curr Biol 2018; 28: 2145–52. 10.1016/j.cub.2018.05.025 29937348 PMC6039257

[210] Herremans AHJ, Hijzen TH, Welborn PFE et al. Effects of infusion of cholinergic drugs into the prefrontal cortex area on delayed matching to position performance in the rat. Brain Res 1996; 711: 102–11. 10.1016/0006-8993(95)01404-7 8680852

[211] Wang Y, Yu T, Yuan C et al. Effects of propofol on the dopamine, metabolites and GABAA receptors in media prefrontal cortex in freely moving rats. Am J Transl Res 2016; 8: 2301–8. 27347337 PMC4891442

[212] Nesbit MO, Ahn S, Zou H et al. Potentiation of prefrontal cortex dopamine function by the novel cognitive enhancer d-govadine. Neuropharmacology 2024; 246: 109849. 10.1016/j.neuropharm.2024.109849 38244888

[213] Mander BA, Winer JR, Walker MP. Sleep and human aging. Neuron 2017;94: 19–36. 10.1016/j.neuron.2017.02.004 28384471 PMC5810920

[214] Peter-Derex L, Yammine P, Bastuji H et al. Sleep and Alzheimer’s disease. Sleep Med Rev 2015; 19: 29–38. 10.1016/j.smrv.2014.03.007 24846773

[215] Maggi G, Trojano L, Barone P et al. Sleep disorders and cognitive dysfunctions in Parkinson’s disease: A meta-analytic study. Neuropsychol Rev 2021; 31: 643–82. 10.1007/s11065-020-09473-1 33779875

[216] Liguori C, Placidi F, Izzi F et al. Sleep dysregulation, memory impairment, and CSF biomarkers during different levels of neurocognitive functioning in Alzheimer’s disease course. Alzheimers Res Ther 2020; 12: 5. 10.1186/s13195-019-0571-3 31901236 PMC6942389

[217] Jaussent I, Bouyer J, Ancelin M-L et al. Excessive sleepiness is predictive of cognitive decline in the elderly. Sleep 2012; 35: 1201–7. 10.5665/sleep.2070 22942498 PMC3413797

[218] Tapia AL, Yu L, Lim A et al. Race and sex differences in the longitudinal changes in multidimensional self-reported sleep health characteristics in aging older adults. Sleep Health 2023; 9: 947–58. 10.1016/j.sleh.2023.08.008 37802678 PMC10841494

[219] Merlino G, Piani A, Gigli GL et al. Daytime sleepiness is associated with dementia and cognitive decline in older Italian adults: A population-based study. Sleep Med 2010; 11: 372–7. 10.1016/j.sleep.2009.07.018 20219426

[220] Cavaillès C, Berr C, Helmer C et al. Complaints of daytime sleepiness, insomnia, hypnotic use, and risk of dementia: A prospective cohort study in the elderly. Alzheimers Res Ther 2022; 14: 12. 10.1186/s13195-021-00952-y 35057850 PMC8780361

[221] Tröndle M, Popov T, Pedroni A et al. Decomposing age effects in EEG alpha power. Cortex 2023; 161: 116–44. 10.1016/j.cortex.2023.02.002 36933455

[222] Auer T, Goldthorpe R, Peach R et al. Functionally annotated electrophysiological neuromarkers of healthy ageing and memory function. Hum Brain Mapp 2024; 45: e26687. 10.1002/hbm.26687 38651629 PMC11036379

[223] Finley AJ, Angus DJ, Knight EL et al. Resting EEG periodic and aperiodic components predict cognitive decline over 10 years. J Neurosci 2024; 44: e1332232024. 10.1523/JNEUROSCI.1332-23.2024 38373849 PMC10977020

[224] Dahl MJ, Mather M, Düzel S et al. Rostral locus coeruleus integrity is associated with better memory performance in older adults. Nat Hum Behav 2019; 3: 1203–14. 10.1038/s41562-019-0715-2 31501542 PMC7203800

[225] Liu KY, Kievit RA, Tsvetanov KA et al. Noradrenergic-dependent functions are associated with age-related locus coeruleus signal intensity differences. Nat Commun 2020; 11: 1712. 10.1038/s41467-020-15410-w 32249849 PMC7136271

[226] Giacobbo BL, Özalay Ö, Mediavilla T et al. The aged striatum: Evidence of molecular and structural changes using a longitudinal multimodal approach in mice. Front Aging Neurosci 2022; 14: 795132. 10.3389/fnagi.2022.795132 35140600 PMC8818755

[227] Rieckmann A, Johnson KA, Sperling RA et al. Dedifferentiation of caudate functional connectivity and striatal dopamine transporter density predict memory change in normal aging. Proc Natl Acad Sci USA 2018; 115: 10160–5. 10.1073/pnas.1804641115 30224467 PMC6176586

[228] Chaves-Coira I, García-Magro N, Zegarra-Valdivia J et al. Cognitive deficits in aging related to changes in basal forebrain neuronal activity. Cells 2023; 12: 1477. 10.3390/cells12111477 37296598 PMC10252596

[229] Zarhin D, Atsmon R, Ruggiero A et al. Disrupted neural correlates of anesthesia and sleep reveal early circuit dysfunctions in Alzheimer models. Cell Rep 2022; 38: 110268. 10.1016/j.celrep.2021.110268 35045289 PMC8789564

[230] Xu W, Tan L, Su B‐J et al. Sleep characteristics and cerebrospinal fluid biomarkers of Alzheimer’s disease pathology in cognitively intact older adults: The CABLE study. Alzheimers Dement 2020; 16: 1146–52. 10.1002/alz.12117 32657026

[231] Theofilas P, Ehrenberg AJ, Dunlop S et al. Locus coeruleus volume and cell population changes during Alzheimer’s disease progression: A stereological study in human postmortem brains with potential implication for early-stage biomarker discovery. Alzheimers Dement 2017; 13: 236–46. 10.1016/j.jalz.2016.06.2362 27513978 PMC5298942

[232] Dahl MJ, Kulesza A, Werkle-Bergner M et al. Declining locus coeruleus-dopaminergic and noradrenergic modulation of long-term memory in aging and Alzheimer’s disease. Neurosci Biobehav Rev 2023; 153: 105358. 10.1016/j.neubiorev.2023.105358 37597700 PMC10591841

[233] Nobili A, Latagliata EC, Viscomi MT et al. Dopamine neuronal loss contributes to memory and reward dysfunction in a model of Alzheimer’s disease. Nat Commun 2017; 8: 14727. 10.1038/ncomms14727 28367951 PMC5382255

[234] Cordella A, Krashia P, Nobili A et al. Dopamine loss alters the hippocampus-nucleus accumbens synaptic transmission in the Tg2576 mouse model of Alzheimer’s disease. Neurobiol Dis 2018; 116: 142–54. 10.1016/j.nbd.2018.05.006 29778899

[235] Berry AS, Harrison TM. New perspectives on the basal forebrain cholinergic system in Alzheimer’s disease. Neurosci Biobehav Rev 2023; 150: 105192. 10.1016/j.neubiorev.2023.105192 37086935 PMC10249144

[236] Rodríguez JJ, Noristani HN, Verkhratsky A. The serotonergic system in ageing and Alzheimer’s disease. Prog Neurobiol 2012; 99: 15–41. 10.1016/j.pneurobio.2012.06.010 22766041

[237] Chen S, He L, Huang AJY et al. A hypothalamic novelty signal modulates hippocampal memory. Nature 2020; 586: 270–4. 10.1038/s41586-020-2771-1 32999460

[238] Fronczek R, van Geest S, Frölich M et al. Hypocretin (orexin) loss in Alzheimer’s disease. Neurobiol Aging 2012; 33: 1642–50. 10.1016/j.neurobiolaging.2011.03.014 21546124

[239] Liguori C, Romigi A, Nuccetelli M et al. Orexinergic system dysregulation, sleep impairment, and cognitive decline in Alzheimer disease. JAMA Neurol 2014; 71: 1498–505. 10.1001/jamaneurol.2014.2510 25322206

[240] Liguori C, Chiaravalloti A, Nuccetelli M et al. Hypothalamic dysfunction is related to sleep impairment and CSF biomarkers in Alzheimer’s disease. J Neurol 2017; 264: 2215–23. 10.1007/s00415-017-8613-x 28900724

[241] Roh JH, Jiang H, Finn MB et al. Potential role of orexin and sleep modulation in the pathogenesis of Alzheimer’s disease. J Exp Med 2014; 211: 2487–96. 10.1084/jem.20141788 25422493 PMC4267230

[242] Liu H, Li J, Wang X et al. Excessive daytime sleepiness in Parkinson’s disease. Nat Sci Sleep 2022; 14: 1589–609. 10.2147/NSS.S375098 36105924 PMC9464627

[243] Dodet P et al. Sleep disorders in Parkinson’s disease, an early and multiple problem. npj Parkinsons Dis 2024; 10: 46. 10.1038/s41531-024-00642-0 38424131 PMC10904863

[244] de Castro Medeiros M, Plewnia C, Mendes RV et al. A mouse model of sleep disorders in Parkinson’s disease showing distinct effects of dopamine D2-like receptor activation. Prog Neurobiol 2023; 231: 102536. 10.1016/j.pneurobio.2023.102536 37805096

[245] Happe S, Baier PC, Helmschmied K et al. Association of daytime sleepiness with nigrostriatal dopaminergic degeneration in early Parkinson’s disease. J Neurol 2007; 254: 1037–43. 10.1007/s00415-006-0483-6 17351722

[246] Amara AW, Chahine LM, Caspell-Garcia C et al. Longitudinal assessment of excessive daytime sleepiness in early Parkinson’s disease. J Neurol Neurosurg Psychiatry 2017; 88: 653–62. 10.1136/jnnp-2016-315023 28554959 PMC7282477

[247] Darvas M, Henschen CW, Palmiter RD. Contributions of signaling by dopamine neurons in dorsal striatum to cognitive behaviors corresponding to those observed in Parkinson’s disease. Neurobiol Dis 2014; 65: 112–23. 10.1016/j.nbd.2014.01.017 24491966 PMC4001780

[248] Schumacher J, Kanel P, Dyrba M et al. Structural and molecular cholinergic imaging markers of cognitive decline in Parkinson’s disease. Brain 2023; 146: 4964–73. 10.1093/brain/awad226 37403733 PMC10689921

[249] Crowley SJ, Kanel P, Roytman S et al. Basal forebrain integrity, cholinergic innervation and cognition in idiopathic Parkinson’s disease. Brain 2024; 147: 1799–808. 10.1093/brain/awad420 38109781 PMC11068112

[250] Fronczek R, Overeem S, Lee SYY et al. Hypocretin (orexin) loss in Parkinson’s disease. Brain 2007; 130: 1577–85. 10.1093/brain/awm090 17470494

[251] Stanojlovic M, Pallais Yllescas JP, Vijayakumar A et al. Early sociability and social memory impairment in the A53T mouse model of Parkinson’s disease are ameliorated by chemogenetic modulation of orexin neuron activity. Mol Neurobiol 2019; 56: 8435–50. 10.1007/s12035-019-01682-x 31250383 PMC6842109

[252] Rao Y, Liu ZW, Borok E et al. Prolonged wakefulness induces experience-dependent synaptic plasticity in mouse hypocretin/orexin neurons. J Clin Invest 2007; 117: 4022–33. 10.1172/JCI32829 18060037 PMC2104495

[253] Repantis D, Schlattmann P, Laisney O et al. Modafinil and methylphenidate for neuroenhancement in healthy individuals: A systematic review. Pharmacol Res 2010; 62: 187–206. 10.1016/j.phrs.2010.04.002 20416377

[254] Dauvilliers Y, Plazzi G, Mignot E et al. Oveporexton, an oral orexin receptor 2-selective agonist, in narcolepsy type 1. N Engl J Med 2025; 392: 1905–16. 10.1056/NEJMoa2405847 40367374

[255] Rosenberg PB, Lanctôt KL, Drye LT et al. Safety and efficacy of methylphenidate for apathy in Alzheimer’s disease: A randomized, placebo-controlled trial. J Clin Psychiatry 2013; 74: 810–6. 10.4088/JCP.12m08099 24021498 PMC3902018

[256] Feldman H, Gauthier S, Hecker J et al. A 24-week, randomized, double-blind study of donepezil in moderate to severe Alzheimer’s disease. Neurology 2001; 57: 613–20. 10.1212/wnl.57.4.613 11524468

[257] Birks JS, Harvey RJ. Donepezil for dementia due to Alzheimer’s disease. Cochrane Database Syst Rev 2018; 6: CD001190. 10.1002/14651858.CD001190.pub3 29923184 PMC6513124

[258] Emre M, Aarsland D, Albanese A et al. Rivastigmine for dementia associated with Parkinson’s disease. N Engl J Med 2004; 351: 2509–18. 10.1056/NEJMoa041470 15590953

[259] Klingberg T . Training and plasticity of working memory. Trends Cogn Sci 2010; 14: 317–24. 10.1016/j.tics.2010.05.002 20630350

[260] López-Ortiz S, Pinto-Fraga J, Valenzuela PL et al. Physical exercise and Alzheimer’s disease: Effects on pathophysiological molecular pathways of the disease. Int J Mol Sci 2021; 22: 2897. 10.3390/ijms22062897 33809300 PMC7999827

[261] López-Ortiz S, Lista S, Valenzuela PL et al. Effects of physical activity and exercise interventions on Alzheimer’s disease: An umbrella review of existing meta-analyses. J Neurol 2023; 270: 711–25. 10.1007/s00415-022-11454-8 36342524

[262] Wang K, Li K, Zhang P et al. Mind–body exercises for non-motor symptoms of patients with Parkinson’s disease: A systematic review and meta-analysis. Front Aging Neurosci 2021; 13: 770920. 10.3389/fnagi.2021.770920 36226304 PMC9549381

[263] Teselink J, Bawa KK, Koo GKY et al. Efficacy of non-invasive brain stimulation on global cognition and neuropsychiatric symptoms in Alzheimer’s disease and mild cognitive impairment: A meta-analysis and systematic review. Ageing Res Rev 2021; 72: 101499. 10.1016/j.arr.2021.101499 34700007

[264] Dinkelbach L, Brambilla M, Manenti R et al. Non-invasive brain stimulation in Parkinson’s disease: Exploiting crossroads of cognition and mood. Neurosci Biobehav Rev 2017; 75: 407–18. 10.1016/j.neubiorev.2017.01.021 28119070

[265] Boggio PS, Ferrucci R, Rigonatti SP et al. Effects of transcranial direct current stimulation on working memory in patients with Parkinson’s disease. J Neurol Sci 2006; 249: 31–8. 10.1016/j.jns.2006.05.062 16843494

[266] Biundo R, Weis L, Fiorenzato E et al. Double-blind randomized trial of tDCS versus sham in Parkinson patients with mild cognitive impairment receiving cognitive training. Brain Stimul 2015; 8: 1223–5. 10.1016/j.brs.2015.07.043 26319357

[267] Kandel ER . The molecular biology of memory storage: A dialogue between genes and synapses. Science 2001; 294: 1030–8. 10.1126/science.1067020 11691980

[268] Takeuchi T, Duszkiewicz AJ, Morris RG. The synaptic plasticity and memory hypothesis: Encoding, storage and persistence. Philos Trans R Soc Lond B Biol Sci 2014; 369: 20130288. 10.1098/rstb.2013.0288 24298167 PMC3843897

[269] Cirelli C, Tononi G. Differential expression of plasticity-related genes in waking and sleep and their regulation by the noradrenergic system. J Neurosci 2000; 20: 9187–94. 10.1523/JNEUROSCI.20-24-09187.2000 11124996 PMC6773024

[270] Cunha C, Brambilla R, Thomas KL. A simple role for BDNF in learning and memory? Front Mol Neurosci 2010; 3: 1. 10.3389/neuro.02.001.2010 20162032 PMC2821174

